# Blockade of MIF–CD74 Signalling on Macrophages and Dendritic Cells Restores the Antitumour Immune Response Against Metastatic Melanoma

**DOI:** 10.3389/fimmu.2018.01132

**Published:** 2018-05-23

**Authors:** Carlos R. Figueiredo, Ricardo A. Azevedo, Sasha Mousdell, Pedro T. Resende-Lara, Lucy Ireland, Almudena Santos, Natalia Girola, Rodrigo L. O. R. Cunha, Michael C. Schmid, Luciano Polonelli, Luiz R. Travassos, Ainhoa Mielgo

**Affiliations:** ^1^Department of Molecular and Clinical Cancer Medicine, University of Liverpool, Liverpool, United Kingdom; ^2^Experimental Oncology Unit (UNONEX), Department of Microbiology, Immunology and Parasitology, Federal University of São Paulo (UNIFESP), São Paulo, Brazil; ^3^Laboratory of Computational Biology and Bioinformatics, Federal University of ABC, Santo André, Brazil; ^4^Laboratoire de Biologie et Pharmacologie Appliquées (LBPA), UMR 8113, Ecole Normale Supérieure, Cachan, France; ^5^Chemical Biology Laboratory, Natural and Human Sciences Center, Federal University of ABC, Santo André, Brazil; ^6^Unit of Biomedical, Biotechnological and Translational Sciences, Department of Medicine and Surgery, Universitá degli Studi di Parma, Parma, Italy

**Keywords:** metastatic melanoma, macrophages, dendritic cells, immune response, peptide-based immunotherapy, macrophage migration inhibitory factor, CD74

## Abstract

Mounting an effective immune response against cancer requires the activation of innate and adaptive immune cells. Metastatic melanoma is the most aggressive form of skin cancer. While immunotherapies have shown a remarkable success in melanoma treatment, patients develop resistance by mechanisms that include the establishment of an immune suppressive tumor microenvironment. Thus, understanding how metastatic melanoma cells suppress the immune system is vital to develop effective immunotherapies against this disease. In this study, we find that macrophages (MOs) and dendritic cells (DCs) are suppressed in metastatic melanoma and that the Ig-CDR-based peptide C36L1 is able to restore MOs and DCs’ antitumorigenic and immunogenic functions and to inhibit metastatic growth in lungs. Specifically, C36L1 treatment is able to repolarize M2-like immunosuppressive MOs into M1-like antitumorigenic MOs, and increase the number of immunogenic DCs, and activated cytotoxic T cells, while reducing the number of regulatory T cells and monocytic myeloid-derived suppressor cells in metastatic lungs. Mechanistically, we find that C36L1 directly binds to the MIF receptor CD74 which is expressed on MOs and DCs, disturbing CD74 structural dynamics and inhibiting MIF signaling on these cells. Interfering with MIF–CD74 signaling on MOs and DCs leads to a decrease in the expression of immunosuppressive factors from MOs and an increase in the capacity of DCs to activate cytotoxic T cells. Our findings suggest that interfering with MIF–CD74 immunosuppressive signaling in MOs and DCs, using peptide-based immunotherapy can restore the antitumor immune response in metastatic melanoma. Our study provides the rationale for further development of peptide-based therapies to restore the antitumor immune response in metastatic melanoma.

## Introduction

Cutaneous melanoma is a cancer that develops from melanocytes generally located in the epidermal basal cell layer of the skin. At very-early stages, single skin lesions can be promptly excised and the 5-year survival rate of melanoma is 98%. Beyond these stages, however, melanoma can metastasize to distant organs including lungs, liver, bones, and brain, and the 5-year survival rate in stage IV drastically decreases to 15–20% (1, 2). The aggressiveness of melanoma is associated with a strong burden of somatic mutations (3), with different neoepitopes making melanoma cells immunogenic and boosting the immune response (4, 5). To evade the immune response, melanomas often activate negative immune checkpoint regulators (ICRs) such as PD-1 and PD-L1 or CTLA-4 that inhibit effector T cell and function in peripheral tissues or lymph nodes, respectively (6, 7). Inhibition of the ICRs with anti-PD-1 and anti-CTLA-4 antibodies enables T-cell-mediated killing of melanoma cells and significantly improved patient outcomes in recent years (5). However, immune checkpoint inhibitors (ICI) are only effective if effector T cells infiltrate the tumor. The generation of effector T cells requires the activation and function of antigen-presenting cells (APCs), such as dendritic cells (DCs) and macrophages (MOs) (8, 9). DCs and MOs are cells from the innate immune system that are essential for starting and shaping the immune response against any damaged tissue, including cancer (7, 10).

Tumor-associated macrophages (TAMs) are one of the most predominant immune cells in melanomas, and the number of TAMs inversely correlates with patients’ outcome, in both early and late stages of melanoma (11). MOs can be polarized into M1-like antitumorigenic and M2-like immunosuppressive MOs (12). We and others have shown that, in tumors, MOs are often polarized into M2-like MOs that support tumor cell proliferation, survival, metastasis, resistance to therapy, and suppress the antitumor immune response (12–16). Similarly, DCs can also acquire immunogenic or tolerogenic behaviors depending on their maturation status (17). Immunogenic DCs support T cell activation and function (17, 18). However, immunogenic DCs often switch into a tolerogenic phenotype during cancer progression, which inhibits the activation and function of effector T cells (19, 20). Tumor cells contribute to the establishment of an immunosuppressive environment by secreting factors that polarize MOs into M2-like immunosuppressive MOs and suppress DCs immunogenic functions leading to Ref. (7, 16, 21). Thus, understanding how metastatic melanoma suppresses the immune system is vital for the development of therapies that restore an effective antitumor immune response.

Bioactive peptides based on immunoglobulin complementary determining regions (CDRs) are promising candidates for adjuvant cancer therapy and can stimulate the innate immune system (22–24). We have previously shown that different CDR peptides display antitumor activities against melanoma and are able to regulate receptors and transcription factors on both tumor cells and immune cells (24–28). Recently, we identified the C36 V_L_ CDR1 peptide (C36L1) as an antitumor CDR-based peptide that inhibits metastatic melanoma cells proliferation and growth *in vitro* and *in vivo* (24, 25). However, the mechanism by which C36L1 inhibits metastatic melanoma progression in a syngeneic model remains unknown.

In this study, we found that C36L1 inhibits metastatic melanoma only in mice that have a competent immune system. C36L1 supports M1-like antitumorigenic MOs and restores DCs pro-inflammatory phenotype and immunogenic function. C36L1 activation of MOs and DCs results in a significant increase in the infiltration of effector T cells in the metastatic lungs, leading to a marked decrease in the tumor burden.

Macrophage migration inhibitory factor (MIF) is an inflammatory cytokine and an important regulator of the innate immune system. Previous studies have shown that MIF can induce an immunosuppressive environment that supports melanoma progression (29, 30). However, the mechanisms by which MIF suppresses the immune cells remain poorly understood. CD74 is the main receptor for MIF. CD74 is the invariant chain of the MHC class II and plays an important role in antigen presentation. CD74 is highly expressed in APCs such as MOs and DCs (31, 32). Thus, MIF and CD74 are emerging attractive targets for immunotherapy.

In this study, we show that the C36L1 peptide binds to CD74 in both MOs and DCs, disturbing its structural dynamics and inhibiting the MIF–CD74 signaling and the immunosuppressive effect on MOs and DCs. These findings highlight the MIF–CD74 axis as an important mechanism of MO and DC immunosuppression in metastatic melanoma, and provide a rationale for further evaluation of CDR-based peptides as therapeutic agents able to restore MOs and DCs’ antitumor functions in metastatic melanoma.

## Materials and Methods

### Cell Culture

Murine melanoma B16F10 cells were cultured in complete RPMI-1640 medium (Thermo Fisher, Waltham, MA, USA) supplemented with 10 mM *N*-2-hydroxyethylpiperazine-*N*2-ethane sulfonic acid (HEPES), 24 mM sodium bicarbonate, 40 mg/L gentamicin, pH 7.2, and 10% fetal bovine serum (FBS), at 37°C. Primary MOs and myeloid DCs were generated from C57BL/6 mice bone marrows and cultured in complete Dulbecco’s Modified Eagle Medium (DMEM) (Thermo Fisher) supplemented with M-CSF1 (10 ng/mL) and RPMI-1640 medium supplemented with GM-CSF (50 ng/mL) and IL-4 (25 ng/mL), respectively. Cultures were regularly checked for contamination.

### Mice and *In Vivo* Metastatic Melanoma Studies

6- to 8-Week-old healthy male C57BL/6 [wild type (WT)] or NOD/Scid/IL-2rγ^null^ (NSG) mice (*n* = 5, per group) were intravenously challenged with 5 × 10^5^ (for WT) or 5 × 10^4^ (for NSG) syngeneic B16F10 viable cells in 0.1 mL of RPMI medium without FBS, and treated on the next day with intraperitoneal doses of 300 µg (10 mg/kg) of C36L1 peptide, for five consecutive days, or with control vehicle (PBS). After 14 days, mice were euthanized, and lungs were harvested and assessed for metastatic colonization. The number of metastatic lesions was quantified using a stereo microscope (magnification, 4×) (Nikon, Tokyo).

### Peptides

Peptides were purchased from Peptide 2.0 (Chantilly, VA, USA). C36L1 peptide (KSSQSVFYSSNNKNYLA-NH_2_) and the irrelevant iCDR control peptide (CE48-H2, INSGGGGTYYADSVKG-NH_2_) were synthesized with an amide group in the C-terminus, at 95–98% purity, determined by high-performance liquid chromatography using a C18 column and subsequently analyzed by mass spectrometry.

### Tissue Paraffin Immunofluorescence

Deparaffinization and antigen retrieval were performed in mouse melanoma lung metastasis using a PT-link system (Dako) and stained as previously described (13) The following antibodies were used for immune stainings: anti-iNOS, anti-CD206, anti-CD103, anti-Ki67, anti-granzyme B, anti-MPO, anti-CD86, anti-CD68, anti-MHC-II, anti-CD11b, anti-Ly6C, anti-Ly6G, and anti-PD-L1 all purchased from Abcam; anti-CD11c and anti-F4/80, purchased from BioLegend; anti-Foxp3 (Cell Signaling); anti-Arg1 (Bioss) and anti-CD8 (Dako) primary antibodies, anti-CD4 (BioLegend) and anti-CD25 (R&D Systems) followed by fluorescently labeled secondary antibodies. Images were acquired using an Axio Observer Light Microscope with the Apotome.2 (Zeiss). Metastatic melanoma lesions were gated by generating a region of interest (ROI), and threshold merge fluorescence was limited to ROI and calculated using the NIS-Elements Advanced Research 4.0 software (Nikon, Tokyo).

### Flow Cytometry Analysis

Lungs from C36L1-treated and control mice were digested in collagenase A and purified for CD11c^+^ DCs using a magnetic bead affinity chromatography approach (Miltenyi Biotec, Woking, UK). Both enriched CD11c^+^ and CD11c^−^ cell fractions were used for DCs and lymphocyte analysis, respectively. DCs were stained with anti-CD11c (V450), anti-CD86 (PE-Cy7), anti-MHC-II (V500), and anti-CD197 (PERCP-CY5.5). Tumor-infiltrating lymphocytes were characterized using anti-CD3 (PE), anti-CD4 (FITC), anti-CD8 (FITC), and anti-NK1.1 (FITC). To analyze splenic Treg cells and MOs, fresh spleens were obtained from mice after treatments and probed with the following conjugated antibodies: anti-CD4 (FITC) and anti-Foxp3 (APC) for lymphocyte analysis, anti-F4/80 (FITC), anti-CD86 (PE-Cy7), and anti-CD40 (APC) for MO analysis. All antibodies were purchased from BD Pharmingen (Franklin Lakes, NJ, USA). Samples were analyzed by flow cytometry using a FACSCanto II (Becton Dickinson, San Jose, CA, USA). Acquired data were analyzed using the FlowJo V10 software (TreeStar Inc., Ashland, OR, USA).

### TGF-β ELISA Assay

CD11c^+^ DCs (1 × 10^5^) were purified from lymphoid tissues of C36L1-treated mice and control vehicle (PBS) using the mouse Pan Dendritic Cell Isolation Kit according to manufacturer’s instructions (Miltenyi Biotec, Bergisch Gladbach, Germany). Primary myeloid DCs were cultured for 48 h at 37°C, and the supernatant was collected for TGF-β quantification using the mouse-TGF-beta ELISA Set (BD, OptEIA™) detection kit according to the manufacturer’s instruction.

### Tumor-Conditioned Medium (TCM) Preparation

B16F10 melanoma cells were cultured in 175 cm^2^ culture flasks and in complete RPMI-1640. When cells reached 70% of confluence, the medium was harvested, filtered for functional assays or concentrated using StrataClean Resin (Agilent Technologies) for MIF detection by immunoblot. Alternatively, to increase the concentration of tumor-secreted factors, B16F10 cells were subcultured in TCM and fresh media (v/v).

### Generation of Bone Marrow-Derived MOs and Myeloid DCs

Bone marrow cells were isolated from the femurs of C57BL/6 mice in cold MAC buffer (Ca^2+^, Mg^2+^ free PBS + 2 mM EDTA + 0.5% BSA), centrifuged at 1,200 rpm for 10 min, resuspended in 5 mL RBC Lysis Buffer (1×, BD Pharm Lyse) and incubated for 5 min at RT. Reaction was terminated in PBS, and cells were centrifuged at 1,200 rpm for 10 min at RT. Cells were resuspended in 5 mL of MAC buffer and carefully added in the top of 5 mL of Histopaque solution (Sigma-Aldrich) in 15 mL tubes and centrifuged at 1,200 rpm, 25 min at 15°C without brake and one acceleration. The monocyte-enriched fraction was collected in a new tube and washed in PBS. Monocytes were further incubated with M-CSF-1 (10 ng/mL) in complete DMEM media (Thermo Fisher) to generate MOs (13), or GM-CSF (50 ng/mL) plus IL-4 (25 ng/mL) in complete RPMI to generate myeloid DCs (17, 33). To generate macrophage-conditioned media (MCM) for the experiment described in Figure 6, MOs were incubated with TCM, MIF (200 ng/mL) or left untreated, in the presence or absence of C36L1 peptide (200 µM) for 72 h, and further incubated in serum-free medium for 48 h. Then, the medium was harvested, centrifuged, and filtered for functional assays or stored at −20°C.

### CD8^+^ T Cells Isolation From Naïve Splenocytes

Lymphocytes were obtained from fresh spleens of naïve mice. The negative CD8a^+^ T Cell Isolation Kit (Miltenyi Biotec, Woking, UK) was used to purify CD8^+^ naïve lymphocytes as per manufacturer’s instructions.

### Flow Cytometry Analysis of Primary DCs

For flow cytometry analysis of primary myeloid DCs, cells were harvested from cultures and blocked with PBS/BSA 1% plus TruStain fcX anti-mouse CD16/32 (BioLegend) and stained using the following conjugated antibodies: DCs: anti-CD11c (APC), anti-CD11b (FITC), anti-MHC-II (Percp-Cy5.5), anti-CD80 (PE-Cy7), and anti-CD86 (PE), all purchased from BioLegend. Stained cells were acquired using Attune™ NxT Acoustic Focusing Cytometer (Thermo Fisher). Data analysis was performed using FlowJo software (Tree Star, Ashland, OR, USA).

### Immunofluorescence and Confocal Microscopy

Fluorescence microscopy of B16F10 cells was performed using the following antibodies: rabbit anti-MIF antibody (Abcam) and secondary antibody solution (anti-rabbit IgG Alexa Fluor 488 (Abcam) and 10 µg/mL of Hoechst 33342). Confocal microscopy for detection of CD74 interaction with C36L1 was performed using a biotinylated C36L1. Briefly, tumor cells were incubated with C36L1 (300 µM) and stained using primary mouse-anti-CD74 (Abcam) and a secondary anti-mouse IgG Alexa Fluor 488 (Abcam) (Green) and Hoechst 33342 (Blue) (Sigma-Aldrich). Streptavidin-Alexa Fluor 594 (red) (Life Technology) was used to probe biotinylated C36L1. Fluorescence and confocal Imaging was performed using an Axio Observer Fluorescence Microscope with the Apotome.2 (Zeiss) and a confocal Zeiss LSM 780 microscope with the 63× 1.4 NA objective, respectively. Colocalization analysis was performed using ImageJ software.

### Primary MOs and Myeloid DCs Culture Assays

Primary MOs and myeloid DCs were generated as described earlier. 5 × 10^5^ cells were seeded in 12-well plates in complete fresh media, and 200 µM of C36L1 peptide was added to the cultures for at least 6 h before the addition of B16F10 TCM or 200 ng/mL of recombinant MIF (R&D System, Minneapolis, MN, USA). Cells were incubated at 37°C for 72 h and further used in FACs analysis for phenotyping or functional assays.

### DC Stimulation for CD8^+^ T Cell Activation Assays

Primary myeloid DCs incubated with C36L1 (200 µM) peptide for 6 h before incubation with recombinant MIF at 200 ng/mL for 72 h. Cells were treated with 200 μM of the tyrosinase-related protein 1 peptide (NDPIFVLLH) as an MHC class I related melanoma antigen. CD8^+^ T cells previously incubated with 30 U/mL of IL-2 and anti-CD3/CD28 dynabeads (Thermo Fisher) were cocultured for 5 days with myeloid DCs in the presence of 30 U/mL of IL-12 (PeproTech, London, UK). CD8^+^ T cells were harvested and cocultured with B16F10 melanoma cells (10:1) for 72 h. CD8^+^ T cells were removed from cultures, and remaining viable B16F10 cells were quantified with a Neubauer chamber using the Trypan Blue dead cells exclusion stain and the MTT colorimetric based assay.

### B16F10 Proliferation Assay With MCM

To obtain different MCM, primary MOs were cultured in the following conditions for 72 h: (1) alone, (2) in the presence of TCM or with recombinant MIF (200 ng/mL), and (3) pre-incubated for 6 h with C36L1 peptide (200 µM) followed by TCM or MIF (200 ng/mL) incubation. Next, the medium was removed, and MOs were further cultured with serum-free medium for 48 h to produce MCM corresponding to the different conditions (MCM1, MCM2, and MCM3). MCM was harvested from the different MO culture conditions, filtered through 0.45 µm and added to 2 × 10^3^ B16F10 melanoma cells plated in 96-well plates stained with CFSE (Thermo Fisher). B16F10 melanoma cells were cultured with the different MCMs for 72 h. Next, B16F10 cells were harvested from wells, stained with propidium iodide (10 µg/mL), and the total number of viable (PI^−^) and proliferating cells (CFSE^−^) was quantified by flow cytometry acquiring fixed volumes of cell suspension using an Attune Flow Cytometer.

### Quantitative Real-Time PCR (qPCR) Experiments

Total RNA from primary MOs previously stimulated with C36L1 (200 µM) for 6 h and TCMs from B16F10 melanoma cells or recombinant MIF (200 ng/mL) for 72 h was isolated using the RNeasy^®^ Mini Kit (Qiagen, Hilden, Germany). cDNA was prepared from 100 ng RNA per sample, and qPCR was performed using gene-specific QuantiTect Assay primers (Qiagen) following the manufacturer’s instructions. qPCR reactions were performed using FIREPol^®^ EvaGreen^®^ qPCR Mix Plus ROX (Solis Biodyne, Tartu, Estonia) in a MaxQuant system. The following primers were used: TGF-β (Mm_Tgfb1_1_SG, Qiagen), IL-10 (Mm_ IL10_1_SG, Qiagen), PD-L1 (Mm_Pdcd1Ig1 _1_SG, Qiagen), Arginase-1 (Mm_ Arg1_1_SG, Qiagen), IL-6 (Mm_Il6_1_SG, Qiagen), and GAPDH (Mm_Gapdh_3_SG, Qiagen). Relative expression levels were normalized to *Gapdh* expression according to the formula $<2^{-\left(C_{{\text{t}}_{\text{gene of interest}}}-C_{{\text{t}}_{\text{gapdh}}}\right)}$ (13) and displayed as fold change units.

### Protein Extraction and Immunoblotting

Primary MOs and myeloid DCs were serum starved for 24 h, treated with C36L1 (200 µM) for 6 h (or left untreated) and stimulated with recombinant MIF (200 ng/mL) at different time points for determination of AKT and ERK1/2 phosphorylation. Protein lysates were separated by electrophoresis, and immunoblotting analyses were performed for: total AKT, total p44/42 MAPK (ERK1/2), phospho-AKT (Ser473), and phospho-ERK1/2 (Thr202/Tyr204). HRP-conjugated secondary antibodies were used, followed by incubation with the ECL substrate (Pierce). All primary and secondary antibodies were purchased from Cell Signaling Technologies (Beverly, MA, USA). Anti-GAPDH (Sigma) was used as protein loading control. To assess the presence of MIF in the TCM, TCM was filtered with 0.45-µm filter and concentrated using StrataClean Resin (Agilent Technologies), and immunoblotted for MIF (Abcam). Phosphorylation ratios were quantified using ImageJ gels’ algorithm, normalized to untreated control lanes.

### Peptide/Protein Binding Prediction

The computational modeling platform PepSite 2.0 (Russel-Lab) (34) was used to predict the binding probability of peptides to mouse MIF (PDB: 1MFI, chain B) and mouse CD74 (PDB: 1IIE, chain B) proteins. Results are displayed as *p* values, where *p* ≤ 0.05 values are the statistically significant binding predictions. iCDR peptide was used as a negative peptide control. Binding probability was calculated using the interval 0.01 < *p* < 0.05, where *p* = 0.01 represents 100% of binding probability and *p* > 0.05 represents 0% of binding probability.

### C36L1 Preparation and Molecular Dynamics (MD)

We obtained the 3D structure of C36L1 by performing *de novo* structure prediction in Pep-Fold3 web server. To perform molecular docking experiments, we carried out an MD simulation on GROMACS 5.1 using CHARMM36 force field. We set up the simulation system on CHARMM-GUI web server. We clustered the MD trajectory to obtain a diverse conformational population to perform molecular docking. All MD frames fitted the reference structure and clustered with GROMOS method by using GROMACS 5.1, with a backbone root-mean-squared deviation cutoff of 5.0 Å for C36L1, resulting in eight different clusters. The center structure of each cluster was used in docking simulations.

### CD74 Normal Mode Calculations and Generation of Low-Energy Conformations

The CD74 structure 1IIE (35) (residues from 118 to 176) was used to perform normal modes analysis using CHARMM c41b1 and CHARMM36 force filed using DIMB module. A distance-dependent dielectric constant was employed to treat the electrostatic shielding from solvation. The five lowest-frequency normal modes were computed as directional constraint to generate low-energy conformers along the mode trajectory using the VMOD algorithm in CHARMM, as previously described (36, 37). The restraints were applied only on Cα atoms, and the energy was computed for all atoms. The structures were displaced from −3.0 Å to +3.0 Å using steps of 0.1 Å, resulting in 61 intermediate energy relaxed structures along each mode.

### Molecular Docking

Molecular docking simulations were performed using iATTRACT algorithm depicting conformational selection and induced fit between both partners. Various conformations of both receptor and ligand (ensemble docking) were simultaneously combined among interface flexibility and rigid body optimizations during docking energy minimization. The best 50 solutions were written for each combination. BINANA 1.2 was used to investigate the specific molecular basis guiding the interaction between CD74 and C36L1.

### Chemiluminescent Dot-Blot Binding Assay

Interaction between the peptide C36L1 and recombinant CD74 was determined by chemiluminescent dot blotting carried out as previously described (24). Briefly, 25 nmol of C36L1 and the irrelevant CDR peptide control (iCDR) and vehicle (0.025% DMSO in dH_2_O) were immobilized on nitrocellulose membranes, blocked and incubated with 25 nM of recombinant CD74 (Abcam) overnight at 4°C. Membranes were washed and incubated with primary mouse anti-CD74 (Abcam), washed and incubated with secondary anti-mouse IgG-HRP (CST). Immunoreactivity was determined using the ECL Western Blotting Substrate (Pierce™), and signal was detected in a transilluminator Alliance 9.7 (Uvitec, Cambridge, UK).

### Statistics

All statistic tests were performed using the GraphPad Prism 5.0 software (San Diego, CA, USA). Statistical differences between experimental and control group were calculated using the Student’s *t*-test. *In vitro* experiments were performed in triplicates. *In vivo* experiment was performed with at least *n* = 5 per treatment group. Sample size for each experiment is described in figure legends. Significant differences are indicated by *p < 0.05, **p < 0.01, and ***p < 0.001.

## Results

### The Antimetastatic Effect of the C36L1 Peptide Requires the Immune System

**Figure 1:**
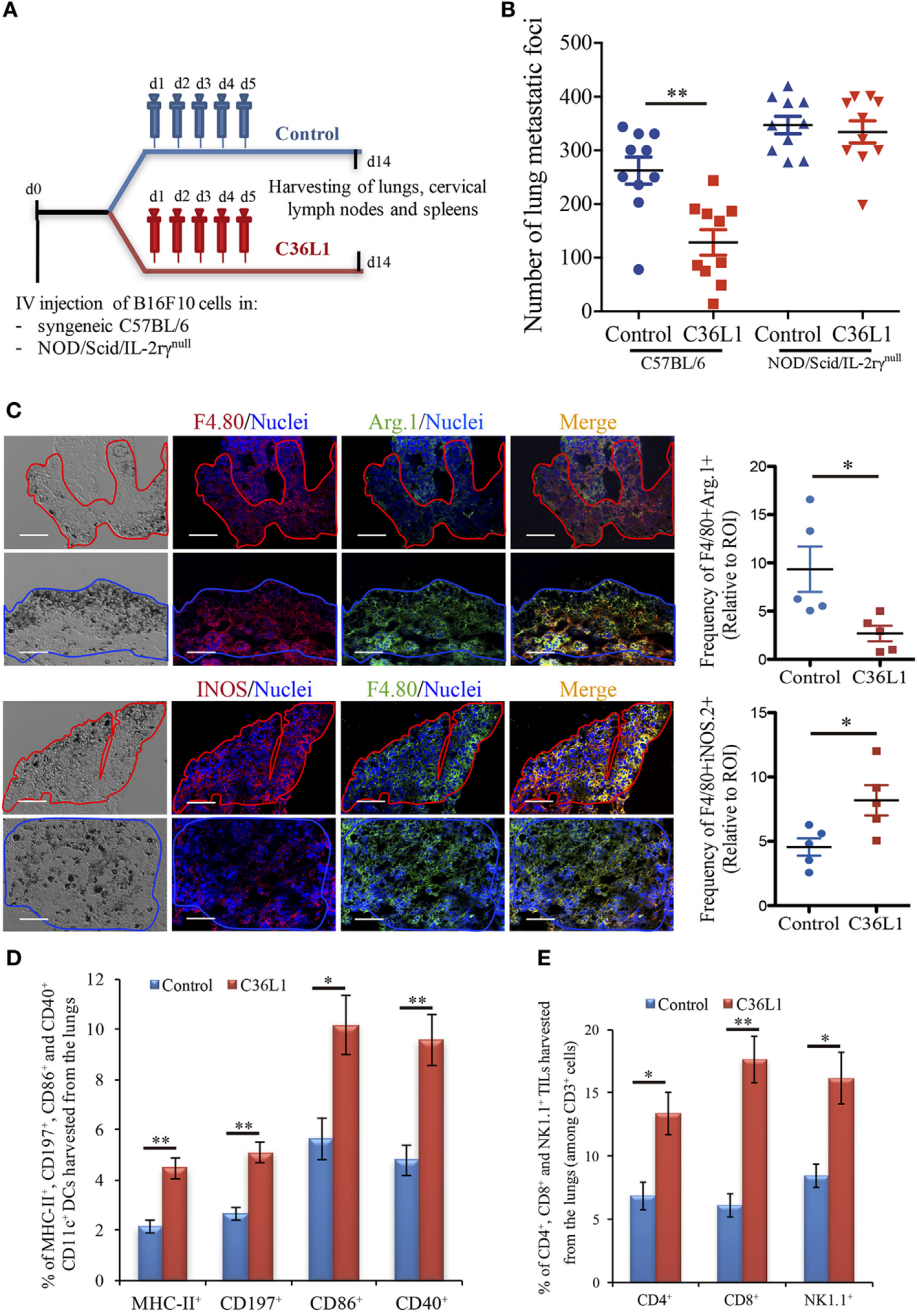
The antimetastatic effect of the C36L1 peptide depends on the immune system. **(A)** Metastatic melanoma model and therapeutic strategy using C36L1 peptide and control vehicle (PBS). At end point, lungs, cervical lymph nodes, and spleens are harvested. **(B)** Number of metastatic foci in immunocompetent (wild type) and immunodeficient (NOD/Scid/IL-2rγ^null^, NSG) mice treated with control vehicle (PBS) or C36L1 peptide. *n* = 10 mice per group (two combined experiments). Values are expressed as means ± SEM and were analyzed using a two-tailed unpaired *t*-test (**p = 0.001). Graph combines two independent experiments. **(C)** Left: Immunofluorescent staining and quantification of F4/80^+^Arg.1^+^ M2-like and F4/80^+^iNOS^+^ M1-like macrophages in lung metastasis from C36L1- and control vehicle-treated mice. Melanoma lung metastatic area appears in dark/brown color in brightfield images. Right; Graphs show quantification of positive F4/80^+^Arg1^+^ (*p = 0.028) and F4/80^+^iNOS^+^ (*p = 0.02) stainings. Nuclei were counterstained with Hoechst 33342 (blue). *N* = 5 mice per group; at least five fields assessed per sample. Values are expressed as means ± SEM and were analyzed using a two-tailed unpaired *t*-test. Blue and red lines indicate the tumor area in C36L1- and control vehicle-treated mice, respectively. Scale bars: 50 µm. **(D)** Flow cytometry quantification of activation markers MHC-II (**p = 0.003), CD197 (**p = 0.002), CD86 (*p = 0.019), and CD40 (**p = 0.007) expressed in CD11c^+^ dendritic cells (DCs) isolated from lungs of C36L1- and control vehicle-treated mice. Data represent quantification of four independent experiments with five pooled lungs per group for each experiment. Values are expressed as means ± SEM and were analyzed using a two-tailed, unpaired *t*-test. **(E)** Quantification of CD4^+^ (*p = 0.03), CD8^+^ (**p = 0.005), and NK1.1^+^ (*p = 0.02) cells among CD3^+^ cells in lung metastatic lesions from C36L1- and control vehicle-treated mice. Bar graphs combine three independent *in vivo* experiments with five pooled lungs per group for each experiment. Values represent means ± SEM and were analyzed using a two-tailed unpaired *t*-test.

We have previously shown that intraperitoneal injections of the antitumor CDR peptide C36L1 significantly decrease pulmonary melanoma metastasis in a syngeneic model (24, 25). In addition, bone marrow-derived myeloid pro-inflammatory DCs displayed equivalent antitumor effect when tumor antigen-primed DCs were pretreated with C36L1 *ex vivo* and adoptively transferred to mice bearing lung melanoma metastasis (24). These findings suggest that the antitumor effects induced by C36L1 *in vivo* may result from the peptide ability to stimulate the host immune response. To further investigate the mechanism of action of C36L1, we treated immunocompetent C57BL/6 and immunodeficient NOD/Scid/IL-2rγ^null^ mice bearing melanoma lung metastasis with C36L1 peptide or control vehicle (Figure 1A). We observed that C36L1 significantly decreased lung metastasis in immunocompetent mice but not in immunodeficient mice (Figure 1B). These findings confirm that C36L1 antitumor effect is driven by its ability to stimulate the immune response against metastatic melanoma.

### C36L1 Restores MOs and DCs Immunogenic Functions in Metastatic Melanoma

Macrophages and DCs are vital for activating effector T cells and shaping the immune response against cancer (7). In solid tumors, including melanomas, MOs and DCs are suppressed by the tumor and lose their ability to activate and support the immune response against cancer (12, 17). TAMs often acquire an M2-like phenotype that hampers the antitumor immune response and supports tumor growth, metastasis, and resistance to therapies (12–14, 38). Similarly, intratumoral DCs often acquire a tolerogenic phenotype and lose their ability to activate effector T cells (17, 39, 40). Thus, effective anticancer immunotherapies must reverse the tumor immunosuppressive environment and restore the immunogenic functions of MOs and DCs. In this respect, we found that C36L1 is able to repolarize M2-like (F4/80^+^CD206^+^Arg1^+^) TAMs into M1-like (F4/80^+^iNOS^+^CD86^+^MHC-II^+^) pro-inflammatory and antitumorigenic MOs (Figure 1C; Figures S1A–C in Supplementary Material). In addition, increased levels of M1-like MOs were also observed in the spleens of C36L1-treated mice, compared with control-treated mice (Figure S5A in Supplementary Material). The number of activated intratumoral DCs (CD11c^+^, MHC-II^+^, CD197^+^, CD40^+^, CD86^+^, and CD103^+^) in metastatic lungs from C36L1-treated mice was significantly increased compared with control-treated mice (Figure 1D; Figure S1D in Supplementary Material). The number of neutrophils and polymorphonuclear myeloid-derived suppressor cells did not significantly change between control- and C36L1-treated metastatic lungs (Figures S3A,B in Supplementary Material). However, we observed a small but statistically significant decrease in the number of monocytic myeloid-derived suppressor cells (Figure S3C in Supplementary Material). C36L1 treatment decreased the secretion of the immunosuppressive cytokine TGF-β by CD11c^+^ DCs from lymphoid organs (spleens and cervical lymph nodes) (Figure S5B in Supplementary Material). These findings suggest that C36L1 repolarizes and reactivates MOs and DCs’ immunogenic and antitumorigenic functions in metastatic melanoma.

### C36L1 Increases the Level of Effector T Cells in the TME

Tumor-specific antigen presentation by DCs and MOs to effector T cells is a crucial step for the generation of an effective immune response against cancer, and increased infiltration of effector T cells in tumors is a good prognostic marker (4, 5). Since treatment with C36L1 decreases melanoma pulmonary metastasis and increases the numbers of pro-inflammatory MOs and DCs, we asked whether C36L1 increases effector T cell infiltration in metastatic tumors. We found that, indeed, C36L1 significantly increased the levels of CD4^+^ T cells from 6.86 to 13.35%, CD8^+^ cytotoxic T cells from 6.11 to 17.6%, and NK1.1^+^ natural killer (NK) cells from 8.44 to 16.13%, in lung metastatic melanoma (Figure 1E; Figure S6A in Supplementary Material). CD8^+^ cytotoxic T cells number and proliferative (CD8^+^Ki67^+^) and activation status (CD8^+^GranzymeB^+^) were significantly increased in C36L1 treated metastatic lungs compared with control-treated lungs (Figures S2A,B in Supplementary Material). We also observed a decrease in the number of regulatory T cells (CD4^+^CD25^+^FoxP3^+^) in metastatic lungs from C36L1-treated mice compared with control mice (Figures S2C,D in Supplementary Material). In lymphoid organs, tolerogenic DCs are responsible for inducing Foxp3^+^ Tregs differentiation by secreting TGF-β. Since C36L1 treatment decreases TGF-β production by DCs (Figure S5B in Supplementary Material), we evaluated whether Tregs were also reduced in lymphoid organs upon C36L1 treatment. Flow cytometry analysis of mice splenocytes revealed a highly significant decrease in the percentage of CD4^+^Foxp3^+^ Tregs from 59.6 to 1.39% following C36L1 treatment (Figure S6B in Supplementary Material). Together, these findings indicate that C36L1 restores DCs and MOs immunogenic functions, increases effector T cell infiltration in metastatic tumors, and inhibits immunosuppressive regulatory T cells.

### C36L1 Inhibits the Suppressive Effects of Tumor-Secreted Factors in MOs

**Figure 2:**
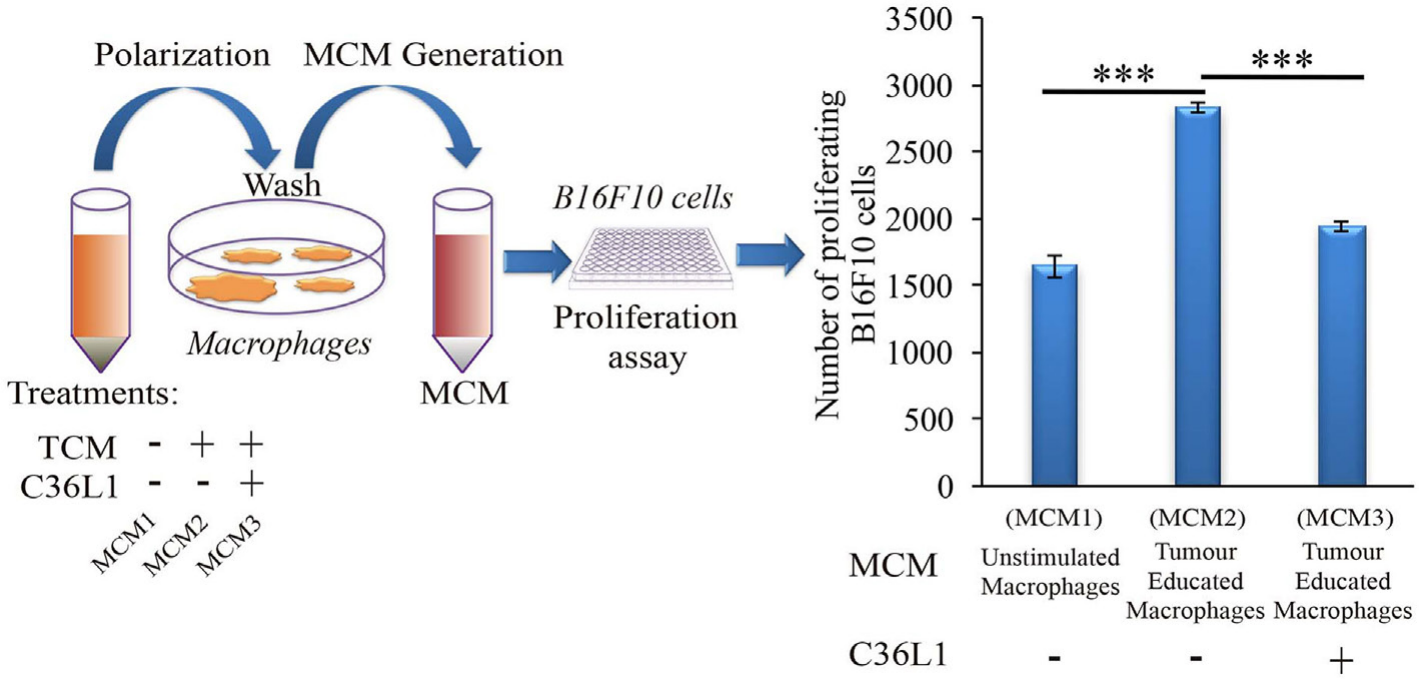
C36L1 counteracts the pro-tumorigenic activity of macrophages (MOs) induced by melanoma derived factors. Left: Schematics describing the workflow of the tumor cell proliferation assay. Tumor cells are exposed to either conditioned media from: untreated MOs (MCM1), MOs exposed to tumor-conditioned media (TCMs) from metastatic melanoma B16F10 cells (MCM2), or MOs exposed to C36L1 peptide + TCM from B16F10 cells (MCM3). Next, macrophage conditioned media (MCM) generated from these three conditions were added into B16F10 melanoma, cells and the number of live proliferating cells was quantified by flow cytometry after 72 h. Right: Bar graph represents average of three independent experiments (*n* = 3). Values represent means ± SEM, and data were analyzed using a two-tailed unpaired *t*-test (***p < 0.001).

Tumor educated MOs exhibit an M2-like phenotype and support cancer progression in several ways, including the direct support of cancer cell proliferation (17). To further understand how C36L1 affects MO function, we cultured metastatic B16F10 melanoma cells with conditioned media from tumor educated MOs (MOs previously exposed to TCMs) in the presence or absence of C36L1. As expected, melanoma cells exposed to tumor educated MOs showed a significant increase in proliferation. Addition of C36L1 abrogated this MO-driven tumor cell proliferation (Figure 2; Figure S6C in Supplementary Material). These results show that MOs exposed to TCMs acquire pro-tumorigenic functions, and this can be inhibited by C36L1 peptide. These findings suggest that C36L1 must interfere with a tumor secreted factor (or its receptor) that regulates MO function.

### C36L1 Binds to MIF Receptor, CD74

**Figure 3:**
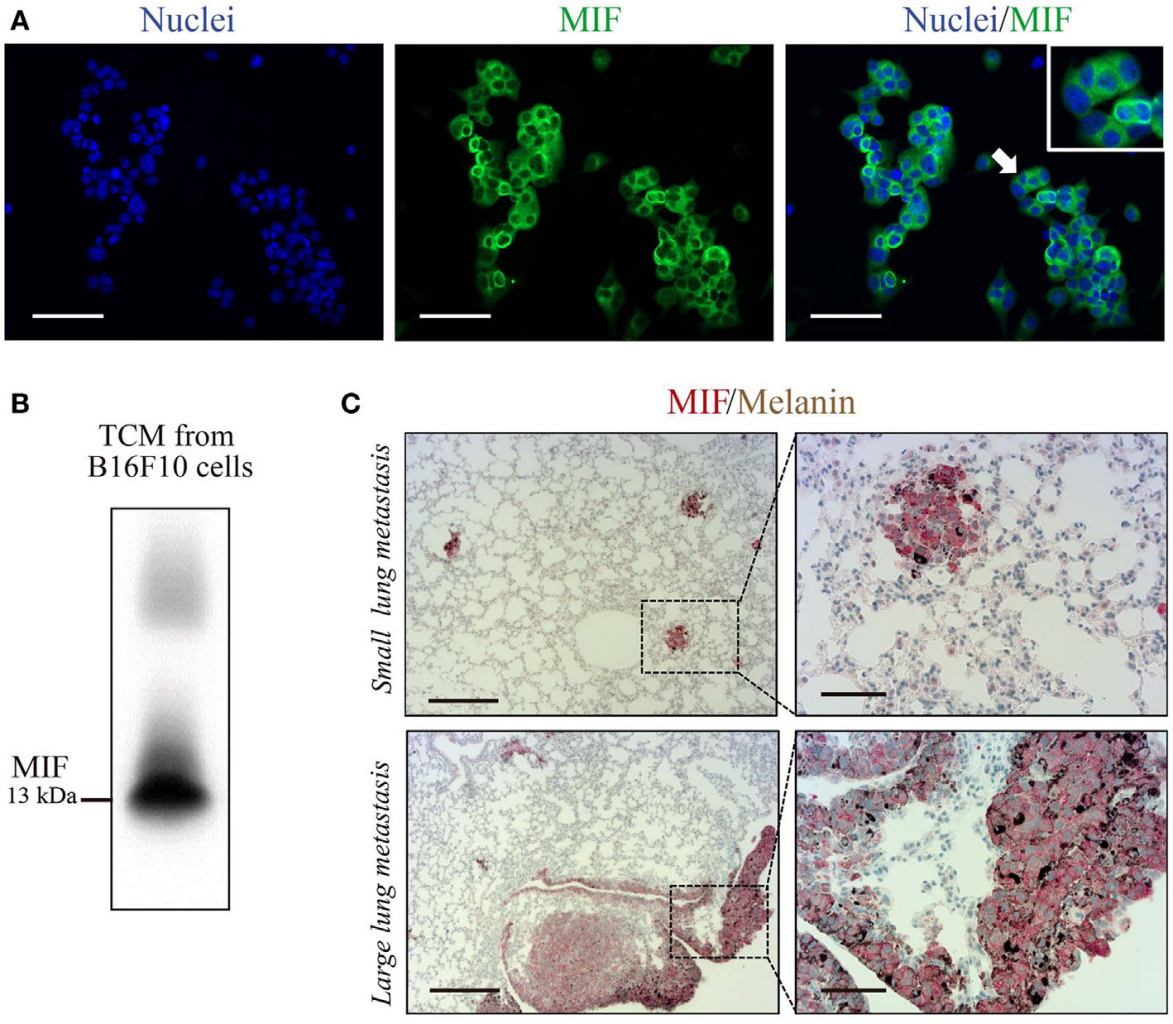
Macrophage migration inhibitory factor (MIF) is secreted by B16F10 metastatic melanoma cells and is highly expressed in lung metastatic lesions. **(A)** Immunofluorescent staining of B16F10 cells stained for MIF (green) and nuclei (blue). Scale bars: 50 µm. **(B)** Immunoblotting analysis of B16F10 tumor-conditioned media (TCMs) detecting secreted MIF. **(C)** Immunohistochemical staining of lung melanoma metastasis showing MIF (in red) in small and large lesions. Dark brown areas are metastatic foci of melanoma cells. Scale bars: 200 µm (left) and 50 µm (right).

C36L1 is a linear and flexible CDR-based peptide. Linear peptides are likely to adopt a few stable conformations and interactive possibilities to different relevant targets (41). Previous studies have shown that stromal and melanoma cells express high levels of MIF, supporting melanoma growth and modulating immune cells in late-stage melanoma (29, 30, 42–46). DCs and MOs both express MIF’s main receptor, CD74 (47). Thus, we hypothesize that C36L1 could interfere with MIF signaling on MOs and DCs. In agreement with previous studies, we observed that B16F10 metastatic melanoma cells express and secrete high levels of MIF *in vitro* (Figures 3A,B), and that MIF is highly expressed in small and large lung metastatic melanoma lesions (Figure 3C).

**Figure 4:**
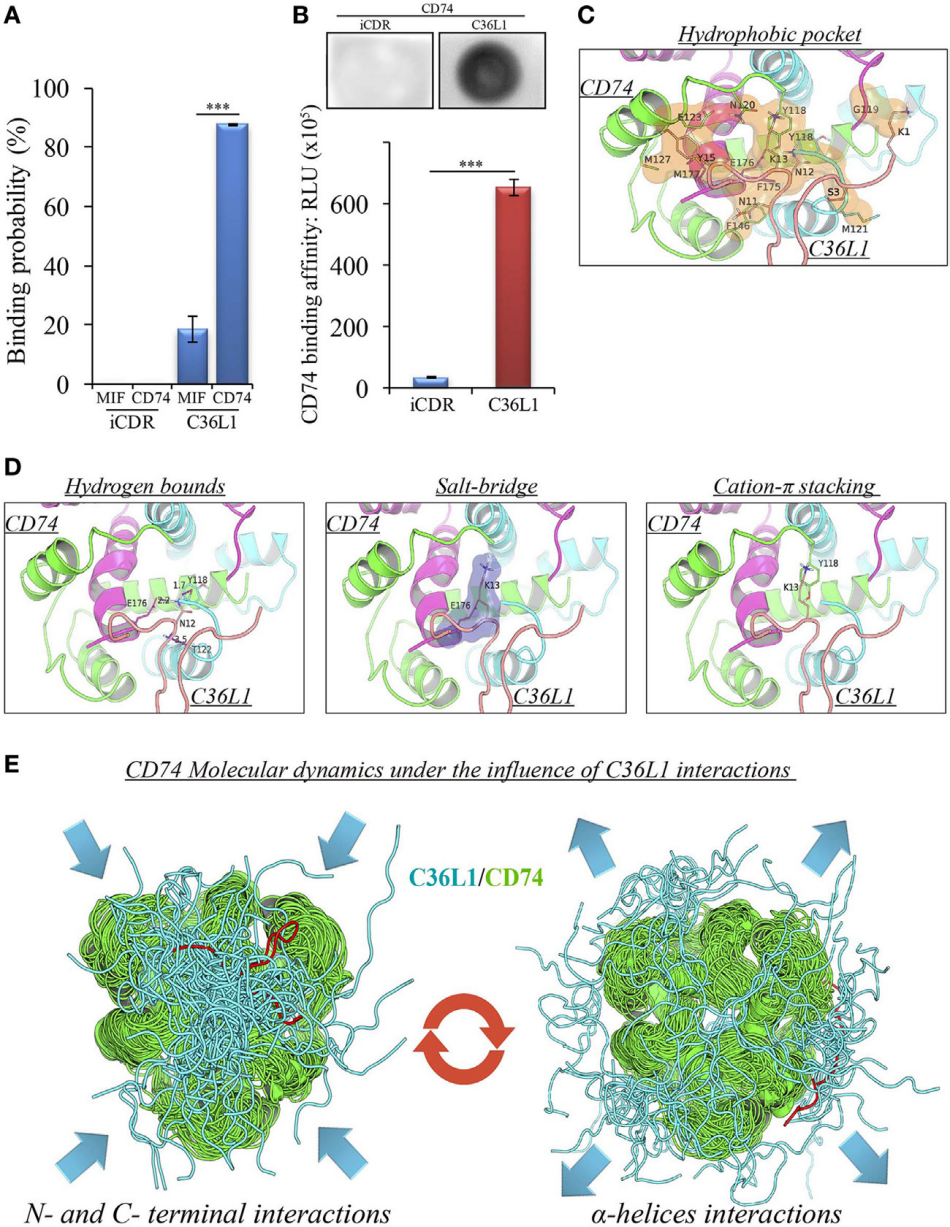
Binding prediction and molecular docking of C36L1 dynamic interactions to macrophage migration inhibitory factor (MIF) and its receptor CD74. **(A)** Binding probability of C36L1 peptide and irrelevant peptide (iCDR) to MIF and its receptor CD74 calculated using PepSite algorithm. Best ranked binding scores (*n* = 5) were included in the analysis for each group (***p < 0.001). **(B)** Dot-blot binding assay for C36L1 and iCDR peptides to mouse recombinant CD74. Bar graph represents mean of RLU in dot area quantified using ImageJ software from triplicates (*n* = 3), ***p < 0.001. **(C)** Hydrophobic pocket (orange) formed by CD74 and C36L1 partners characterized by carbon–carbon interactions above a 4 Å distance cutoff. **(D)** Electrostatic interactions between CD74 and C36L1 peptide: hydrogen bonds formed between partners. Donor–acceptor distances are described; salt bridge formed involving K13; cation–π stacking between tyrosine residues of chain A of CD74 and C36L1. CD74 chains A, B, and C are colored in green, cyan, and magenta, respectively. C36L1 is colored in yellow. **(E)** Overlap of highest and lowest free energy results for C36L1 (cyan) in complex with CD74 (green). Left: Overlap of the lowest free energy 50 poses showing major concentration of C36L1 peptide at the CD74 N- and C-terminal interface. Lowest peptide free energy pose highlighted in red. Right: Overlap of the lowest free energy 50 poses, where C36L1 visits other regions of CD74, including the external region of the α-helices. Lowest peptide free energy highlighted in red.

A pilot study addressing the binding probability of C36L1 to MIF and its receptor CD74 was carried out using the computational modeling prediction of peptide-binding sites to protein surfaces and the PepSite 2.0 algorithm (34). This *in silico* approach predicted a statistically significant binding of C36L1 to mouse CD74 B chain (PDB: 1IIE) protein (*p* < 0.001), and a potential binding to mouse MIF B chain (PDB: 1MFI) protein (*p* = 0.04) (Figure 4A). No interaction with either CD74 or MIF was predicted for an irrelevant control CDR peptide (iCDR—CE48-H2), which was previously observed to have no effect on metastatic melanoma proliferation *in vitro* and progression *in vivo* (25) (Figure 4A; Figure S7A in Supplementary Material). We used the PepSite 2.0 algorithm to identify the amino acid residues involved in the interaction of C36L1 to CD74 and found that the peptide is predicted to interact with Tyr (118), Arg (179), and His (180) residues from the B chain of the murine/human CD74 protein, highlighted in red (Figure S7B in Supplementary Material). Interestingly, Meza-Romero et al. have recently described that some of these residues (highlighted in green) are also critical for the interaction of MIF with the CD74 antagonist (RTL-1000) (48). The *in silico* predicted interaction of C36L1 with CD74 was further confirmed in a dot-blot binding assay using both immobilized C36L1 and iCDR peptides against recombinant murine CD74 protein (Figure 4B). These results suggest that C36L1 could act as an antagonist of MIF, since its interaction occurs on critical binding sites used by MIF to interact with CD74.

To further investigate this, we performed a molecular docking study between C36L1 and CD74 protein. Docking calculations resulted in 122,000 different poses of which the worst 1% was discarded for presenting outliers’ energy values. The average energy of remaining structures was 60.8 kcal/mol, and more than 95% of them presented thermodynamically favorable binding energies (Figure S7C in Supplementary Material). The best solution occurred between C36L1 cluster 5 centroid and a CD74 structure with large opening (2.7 Å from reference) along normal mode 10, which shows an open–close motion. This pose presented −192.6 kcal/mol as free energy of binding and is depicted in Figure S7D in Supplementary Material. The key interaction elements observed in this complex were analyzed using BINANA algorithm. Hydrophobic contacts forming an extended pocket along the interface of all CD74 subunits were observed (Figure 4C). Stronger interactions were also observed: three critical hydrogen bonds, one salt bridge and one cation–π stacking interaction between CD74 and C36L1 peptide (Table 1; Figure 4D). Interestingly, C36L1 cluster 5 centroid appears in 30 of top 50 best poses suggesting that this peptide conformation is likely to be privileged to bind CD74. Moreover, structures with large displacements along mode 10 of CD74 are more frequent; the worst ranked structures were less displaced. C36L1 interacts better with CD74 as it moves according to normal mode 10, whereas once CD74 returns to the relaxed conformation, the peptide-binding affinity decreases and the complex dissociates. Furthermore, the overlap of 50 best solutions showed a putative preferred binding region of C36L1 to the interface formed between N- and C-terminal portions of CD74 monomers. This binding site is confirmed by the observation of C36L1 main binding to CD74 α-helices, only in the worst solutions. In Figure 4E, blue arrows indicate spatial distribution of C36L1 (blue) over CD74 altered structures (green), and the best and worst poses of C36L1 are shown in red. A video representing the consequences of this dynamic interaction between C36L1 and CD74 tertiary structure is shown in Video S1 in Supplementary Material.

**Table 1:** Molecular docking main findings.

Parameters	Residues	
Hydrogen bonds	C36L1	CD74
	ASN12	THR122-B
	ASN12	TYR118-B
	ASN12	GLU176-C

Salt bridges	LYS13	GLU176-C
Cation–π stacking	LYS13	TYR118-A

Scoring iAttract	−192,575 (kcal/mol)	
Hydrophobic contacts	81 (C–C)	

### C36L1 Binds to CD74 on MOs and DCs and Disrupts Downstream Signaling

CD74 is a transmembrane protein mainly expressed in APCs and associated with the MHC II intracellular trafficking. CD74 is the main receptor for MIF in MOs and DCs, and MIF binding to CD74 leads to immunosuppression of MOs, activation of myeloid-derived suppressor cells, suppression of NK cells, and inhibition of T cell activation (29, 43, 47, 49–51). Thus, we evaluated whether C36L1 peptide (as predicted in the *in silico* approach) physiologically binds to CD74 receptor on MOs and DCs.

**Figure 5:**
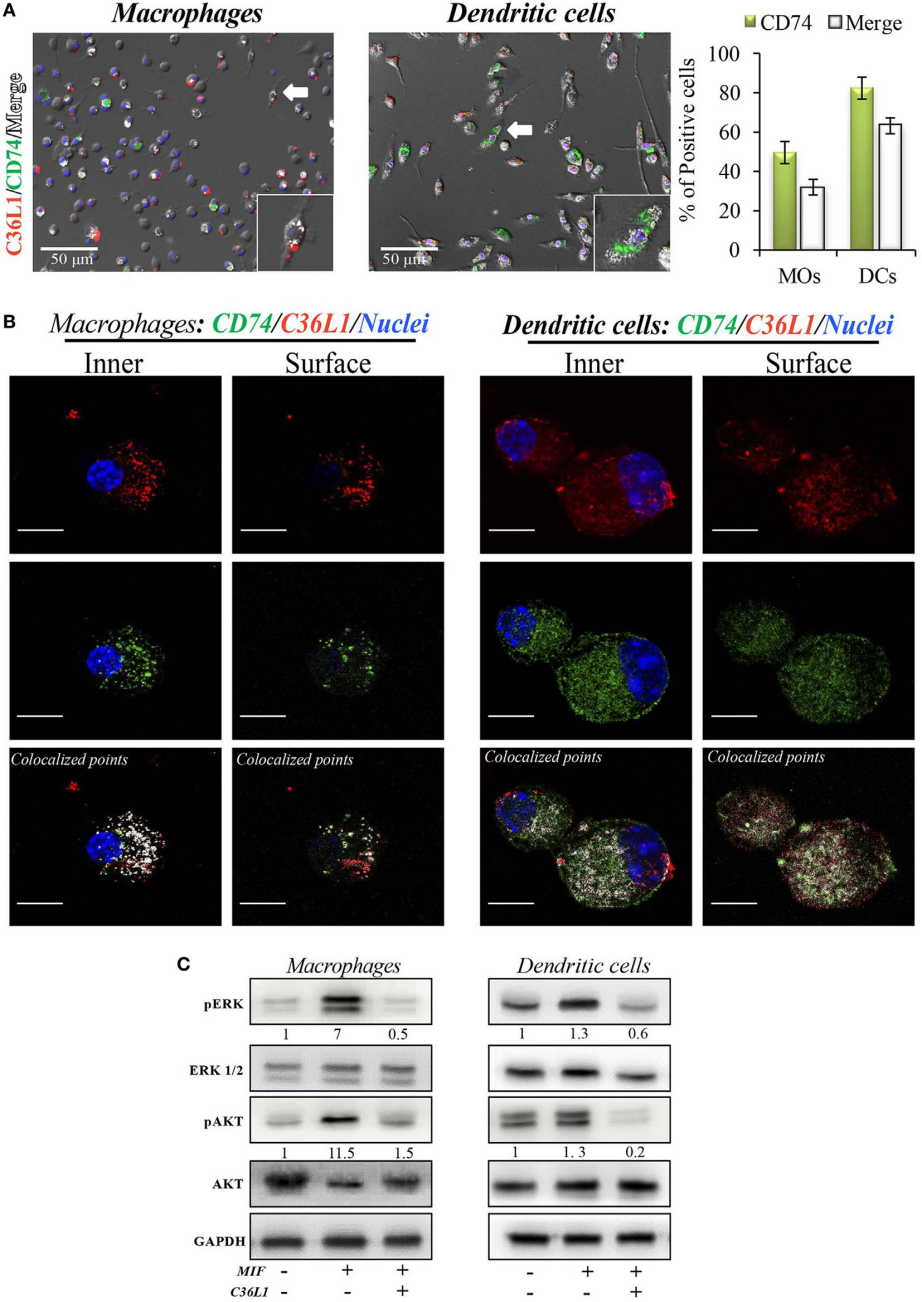
C36L1 interacts with CD74 in both macrophages (MOs) and dendritic cells (DCs) and inhibits macrophage migration inhibitory factor (MIF)/CD74 signaling. **(A)** Immunofluorescent staining of C36L1 (red), CD74 (green), and nuclei (blue) in primary MOs and DCs. CD74 interactions with C36L1 were quantified using automated analysis in ImageJ. Arrows indicate merged channels depicted in white. Four fields per slide were quantified. Scale bars: 50 µm. **(B)** Representative fluorescent confocal microscopy images showing colocalization of C36L1 peptide (red) and CD74 (green) in the intracellular and surface focal plane of both primary MOs (left) and DCs (right). Co-localized points were detected using ImageJ colocalization algorithm, depicted in white. Scale bars: 10 µm. **(C)** Immunoblotting analysis of phosphorylated AKT and ERK1/2 on primary MOs (10 and 20 min, respectively) and DCs (5 min) previously treated with C36L1 (200 µg/mL) or left untreated, and further treated with recombinant MIF (200 ng/mL).

To address these interactions, primary bone marrow-derived MOs and DCs were incubated with biotinylated C36L1 probed with streptavidin-PE (red) and stained for CD74 (green). We observed that C36L1 binds to CD74 in both MOs and DCs (Figure 5A). CD74 can be expressed intracellularly and at the plasma membrane. Using confocal microscopy, we observed that C36L1 co-localizes with CD74 both intracellularly and at the cell membrane (Figure 5B). MIF interaction with CD74 receptor activates different cell signaling pathways, including the PI3K/AKT and the MAPK signaling pathways (47, 49, 52). In agreement with this, we observed that recombinant MIF induces the phosphorylation of AKT (S473) and ERK (Thr202/Tyr204) in both primary MOs and DCs (Figure 5C). However, pre-incubation of MOs and DCs with C36L1 inhibited MIF-induced AKT and ERK downstream signaling on MOs and DCs. These findings show that C36L1 binds to CD74 on MOs and DCs and disrupts MIF–CD74 signaling on these cells.

### C36L1 Inhibits MIF-Induced Suppression of MOs and DCs and Restores Their Immunogenic and Antitumorigenic Functions

**Figure 6:**
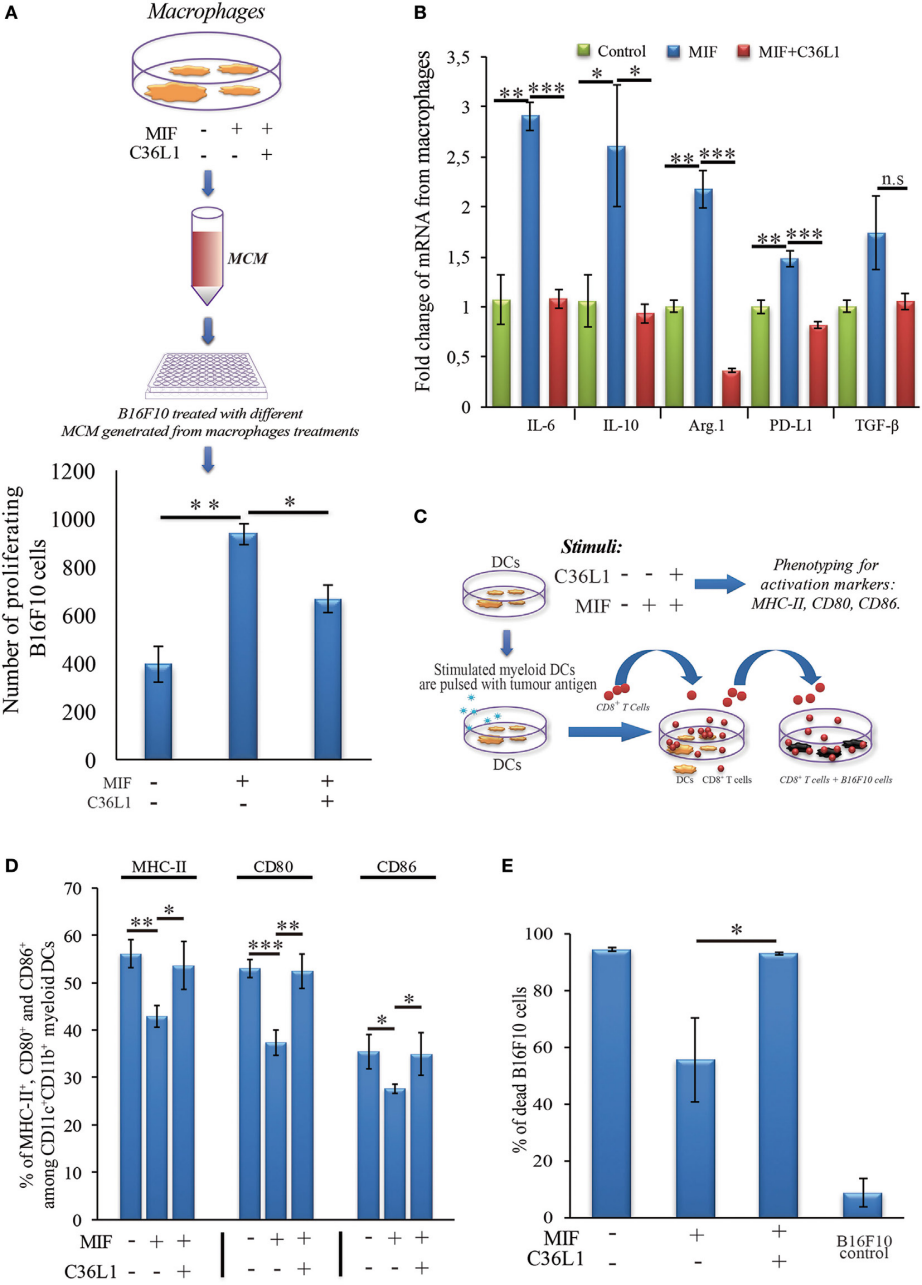
C36L1 blocks macrophage migration inhibitory factor (MIF) induced immunosuppressive effect on macrophages (MOs) and dendritic cells (DCs). **(A)** Top: Schematics describing the workflow of the tumor cell proliferation assay. B16F10 metastatic melanoma cells are exposed to conditioned media from: untreated MOs, MOs exposed to MIF (200 ng/mL), or MOs exposed to C36L1 (200 µg/mL) + MIF (200 ng/mL). The number of live proliferating B16F10 cells was quantified by flow cytometry after 72 h. Bottom: Bar graph represents average of three independent experiments (*n* = 3), mean ± SEM. Data were analyzed using a two-tailed unpaired *t*-test (***p < 0.001). **(B)** C36L1 blocks MIF-induced immunosuppressive effect on primary MOs. mRNA levels of *TGF-β* (n.s. = 0.058), IL-10 (*p = 0.049, *p* = 0.042), Arg.1 (**p = 0.002, ***p < 0.001), PD-L1 (**p = 0.0049, ***p < 0.001), and IL-6 (**p = 0.0015, ***p < 0.001) from MOs exposed to recombinant MIF in the presence or absence of C36L1 peptide. Experiment was performed in triplicates (*n* = 3). Values represent mean ± SEM and were analyzed using a two-tailed unpaired *t*-test. **(C)** Schematics describing the different conditions in which DCs were cultured and then used to activate T cells. Primary DCs were incubated with MIF (200 ng/mL) in the presence or absence of C36L1 peptide and activation markers were quantified by flow cytometry. These DCs were further pulsed with a melanoma antigen peptide and incubated with syngeneic purified CD8^+^ T cells. Next, T cells were harvested and incubated with melanoma B16F10 cells at a ratio of 10/1 CD8^+^ T cells**/**B16F10 tumor cell. **(D)** Quantification of MHC-II (*p* = 0.01), CD80 (*p* < 0.001), and CD86 (*p* = 0.02) activation markers in DCs performed by flow cytometry. Bar graph represents mean ± SEM, from three independent experiments (*n* = 3). Data were analyzed using a two-tailed unpaired *t*-test. **(E)** Bar graph showing the quantification of dead B16F10 cells after incubation with CD8^+^ T cells. Best of three independent experiments is shown, mean ± SEM from biological triplicates, one-tailed unpaired *t*-test (*p = 0.032).

To further understand the mechanism of action of C36L1 on MOs, we evaluated the immunosuppressive and tumor supporting functions of MOs exposed to MIF in the presence or absence of C36L1. MOs exposed to MIF supported the proliferation of melanoma cells (similar to what we observed when we exposed MOs to TCMs in Figure 2). C36L1 treatment abolished this MIF-induced pro-tumorigenic function of MOs (Figure 6A). C36L1 also significantly decreased the expression of the immunosuppressive factors TGF-β, IL-10, IL-6, Arginase-1, and PD-L1 by MOs exposed to MIF (Figure 6B).

To understand the mechanism of action of C36L1 on DCs, we evaluated the expression levels of DC activation markers as well as DCs ability to activate cytotoxic T cells in the presence or absence of MIF and C36L1 (Figure 6C). Treatment of primary myeloid DCs with MIF significantly decreased the levels of the maturation and co-stimulatory markers CD86, CD80 and MHC-II. Treatment with C36L1 peptide counteracted the immunosuppressive effect of MIF on DCs (Figure 6D). DCs ability to activate cytotoxic T cell killing function was also significantly impaired by MIF but rescued by C36L1 treatment (Figures 6C,E; Figure S8 in Supplementary Material). All together, these results provide functional evidence that C36L1 restores DCs and MOs immunogenic and antitumorigenic functions by interfering with the MIF/CD74 immunosuppressive signaling axis.

## Discussion

Cutaneous melanomas are common in the Western hemisphere causing the majority (75%) of deaths related to skin cancer (53). The incidence rate of melanoma increases faster than for any other cancer (52). At very-early stages, melanomas can be surgically removed, and the 5-year survival rate of melanoma is 98%. However, melanoma can metastasize to distant organs including lungs, liver, bones, and brain, and the 5-year survival rate of patients with metastatic melanoma drastically decreases to 15–20% (1, 2). Treatment with ICI has significantly increased the 5-year survival rate of melanoma patients (1, 54), but the number of non-responders is still high, with the lack of response being currently intensively investigated. Mutations of gene families of cytokines, chemokine levels, mesenchymal transition, E-cadherin, and other proteins expressed in tumors are being studied (55). Understanding and targeting the immunosuppressive tumor microenvironment to restore an antitumor immune response is an area of great interest (7, 29, 56, 57). Therefore, understanding the mechanisms by which metastatic melanoma suppresses antitumor immunity could further contribute to the development of new combinatorial agents that restore the immune response against metastatic melanoma.

Synthetic peptides based on immunoglobulin-CDR sequences have shown promising antitumor properties, and some of these peptides display immune stimulatory functions (22, 24–26).

We previously found that the C36 V_L_ CDR1 peptide (C36L1) displays dose-dependent antitumor activities *in vitro* against B16F10 melanoma cells, exerting microtubule de-polymerization at low concentrations and cell death at high concentrations (24). Our *in vivo* studies show that the antitumor effect induced by the C36L1 peptide strictly depends on its original sequence since the shuffled peptide was unable to exert any antitumor effects in the metastatic melanoma setting, and acted in a similar way as the PBS vehicle control (24). We also observed that the antitumor activity of C36L1 is not a general property of Ig-CDRs, since other CDR sequences (i.e., CE48-H2) did not show such antitumor effects (25). Short peptides can interact with more than one ligand, with variable affinities under different conditions or microenvironments. We have previously uncovered peptide sequences that exert different therapeutic activities against infection diseases and cancer (22, 26, 27).

In this study, we uncover the mechanism by which C36L1 restores an effective immune response against metastatic melanoma *in vivo*. We found that C36L1 is able to decrease melanoma metastatic growth in wild-type mice but not in immunodeficient mice, suggesting that *in vivo*, the antitumor effect of C36L1 requires the immune system. Specifically, we found that C36L1 is able to repolarize M2-like immunosuppressive TAMs into immunogenic and antitumorigenic M1-like MOs. C36L1 also promotes the activation and immunogenicity of DCs. C36L1-driven activation of the innate immune system leads to the inhibition of immunosuppressive Tregs, the activation of effector T cells and subsequently to the killing of metastatic melanoma cells. Mechanistically, we found that C36L1 binds to the MIF receptor CD74 on MOs and DCs, thereby inhibiting MIF immunosuppressive effect on these innate immune cells, and shifting the balance from an immunosuppressive tumor microenvironment into a pro-inflammatory immunogenic environment in which the antitumor immune response is reinvigorated.

Tumors, including melanomas, secrete factors that inhibit the immune system. Among these factors, MIF has been recently shown to have immunosuppressive activities, in many cancers, including glioblastoma, breast, pancreatic cancer, and melanoma (29, 30, 49, 58–60). Thus, MIF is an emerging attractive target for immunotherapy. In pancreatic cancer, MIF is an important downstream regulator of fibrosis that culminates in the recruitment of TAMs favoring metastasis (21). In cutaneous melanoma, MIF is produced by melanoma cells to support growth and induce immunosuppression (29, 30, 42–44, 51). However, the role of MIF in metastatic melanoma remains unclear. In glioblastoma, MIF can also induce pro-inflammatory functions, including M1-like MO polarization (58, 61). Bevacizumab, a monoclonal antibody that targets VEGF may also interact and neutralize MIF in glioblastomas, inducing the polarization of MOs into the M2-like phenotype that contributes to therapy resistance (58). This dual and opposite effect of MIF on the immune response depends on the cytokine milieu in the tumor microenvironment and on the levels of MIF. In fact, very low or high concentrations of MIF are thought to suppress the immune response, while intermediate doses rather promote pro-inflammatory and antitumor effects (58).

Different drugs targeting MIF and its main receptor CD74 are in clinical development in many diseases, including cancer (31, 32, 48, 62–65). The MIF inhibitor 4-iPP is so far the only immunomodulatory agent described to be effective in melanoma and has shown promising results in subcutaneous melanoma, associated with an increase in monocyte pro-inflammatory functions (30). The effect of blocking MIF–CD74 signaling in metastatic melanoma has not yet been investigated. Targeting CD74 seems to be a promising anticancer therapeutic strategy to disrupt MIF-induced suppressive signaling effect on monocytes (31, 49, 65). The most well-characterized CD74 inhibitor is Milatuzumab, a monoclonal antibody approved for the treatment of chronic lymphocytic leukemia with acceptable side effects in humans including leukopenia, rash, nausea, and vomiting at low grade (65). In the field of drug discovery, peptide-based approaches emerge with intrinsic advantages, compared with antibodies including their small size, lack of immunogenicity, high affinity, specificity to different targets, low toxicity, good tissue penetration, and biocompatibility (22, 25, 26). Peptides can exert immunomodulatory functions and have been shown to neutralize immune checkpoint receptors in cancer (66–68). Indeed, linear peptides such as CDR peptides are flexible and likely to bind to different biologically relevant targets (41). Ig-CDR peptides, like C36L1, are mostly non-toxic in normal tissues and untransformed cell lines and are short living in the plasma due to proteolysis and renal filtration. However, since they can promptly interact with immune cells such as DCs and MOs, they could modulate the immune response in advanced stage melanomas.

**Figure 7:**
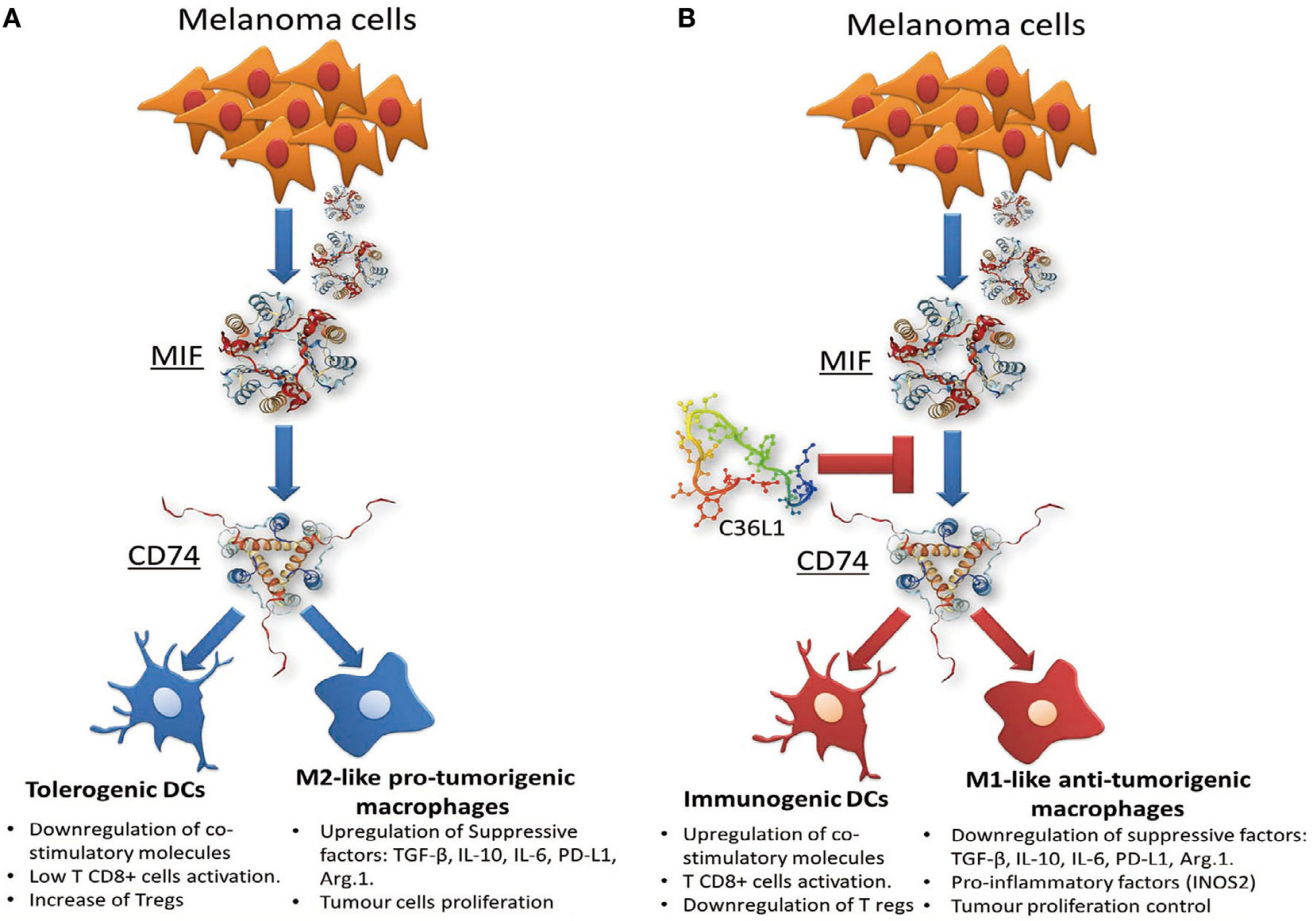
Scheme of the mechanism of action of the C36L1 peptide in macrophages (MOs) and dendritic cells (DCs). C36L1 binds to receptor of macrophage migration inhibitory factor (MIF) CD74, thereby blocking its immunosuppressive effect on MOs and DCs **(A)**, restoring their antitumorigenic functions and their capacity to activate and support an effective immune response against metastatic melanoma **(B)**.

In this study, we found that C36L1 interaction with the CD74 receptor expressed on MOs and DCs is sufficient to inhibit MIF–CD74 signaling and to restore MOs and DCs antitumorigenic functions (Figure 7). Our *in silico* studies show that the flexibility of this linear peptide allows its transient interaction with the CD74 receptor, disturbing its MD in the cell membrane. C36L1–CD74 interaction seems to be crucial to disrupt CD74 interaction with MIF in both MOs and DCs. The cell internalization of CD74 conjugates is a well-known pharmacological characteristic of CD74 (50, 65), which has been recently explored as a drug-carrier strategy for the treatment of lymphomas and B cell malignancies (65). CD74 internalization independent of MIF binding could impair the activation of downstream signaling (31, 69). In this respect, we found that C36L1 binds to CD74 at the cell membrane as well as in the intracellular space of MOs and DCs. This suggests that C36L1 binding to CD74 may promote its cytosolic internalization making it unavailable for binding to MIF. MIF binding to CD74 activates the PI3K/AKT and MAPK signaling pathways, and both these pathways have been related to monocyte immunosuppression, and MO M2-like polarization (45, 47, 49, 52). In agreement with these studies, we found that C36L1 inhibits MIF-induced AKT and ERK1/2 phosphorylation in both primary MOs and DCs and restores their antitumorigenic and immunogenic functions (Figure 7).

In conclusion, our findings suggest that MIF is highly secreted in metastatic melanoma and is an important immunosuppressor of MOs and DCs. Blocking MIF signaling through CD74 on MOs and DCs, using the C36L1 Ig-CDR-based peptide, restores the pro-inflammatory functions of MOs and DCs thereby harnessing the immune response against metastatic melanoma. This study provides a rationale for further evaluation of CDR-based peptides as therapeutic agents to restore the ability of MOs and DCs to start and shape an effective anticancer immune response.

## Ethics Statement

Animal experiments were carried out in accordance with the recommendations of the National Council for the Control of Animal Experimentation (CONCEA, Brazil) and approved by the Ethics Committee of Federal University of São Paulo, registered with the number CEUA No. 7588260915. Weight loss, lethargy, and weakness that could result in inability to feed and drink, as well as infection with systemic signs of illness, were considered as standard clinical symptoms that indicate deteriorating health conditions requiring euthanasia before the end of the experiment.

## Author Contributions

CRF performed most of the experiments. RA performed *in vivo* experiments. SM assisted with flow cytometry experiments. PR-L performed the molecular docking and dynamics studies. LI helped with isolation of primary cells and with methodology development. AS assisted with tissue processing and IHC experiments. NG assisted with *in vivo* and flow cytometry procedures. RC assisted with molecular docking and dynamic analysis of C36L1/CD74 interaction model. MS assisted with methodology development and provided conceptual advice. LP assisted with the initial conception of Ig-CDR peptide biological functions. LT generated the peptide, assisted in its functional characterization, and supervised the *in vivo* experiments. AM and CRF designed experiments and wrote the manuscript. AM supervised the project. All the authors helped with analysis and interpretation of results and approved the manuscript.

## Conflict of Interest Statement

The authors declare that the research was conducted in the absence of any commercial or financial relationships that could be construed as a potential conflict of interest.
