# Supplementary material for: Blockade of MIF–CD74 Signalling on Macrophages and Dendritic Cells Restores the Antitumour Immune Response Against Metastatic Melanoma
Source: Front Immunol. 2018 May 23;9:1132. doi: 10.3389/fimmu.2018.01132 (PMC5974174; doi:10.3389/fimmu.2018.01132)
Supplement: Supplementary file 2 [file presentation_2.PDF]

Supplementary Material

Blockade of MIF-CD74 signalling on macrophages and dendritic cells restores the anti-tumour immune response against metastatic melanoma.

Carlos R. Figueiredo[1,2], Ricardo A. Azevedo[2], Sasha Mousdell[1], Pedro T. Resende-Lara[3,4], Lucy Ireland[1], Almudena Santos[1], Natalia Girola[2], Rodrigo L.O.R. Cunha[5], Michael C. Schmid[1], Luciano Polonelli[6], Luiz R. Travassos[2], Ainhoa Mielgo[1*].

* senior corresponding author

1 Supplementary Data

1.1 Supplementary

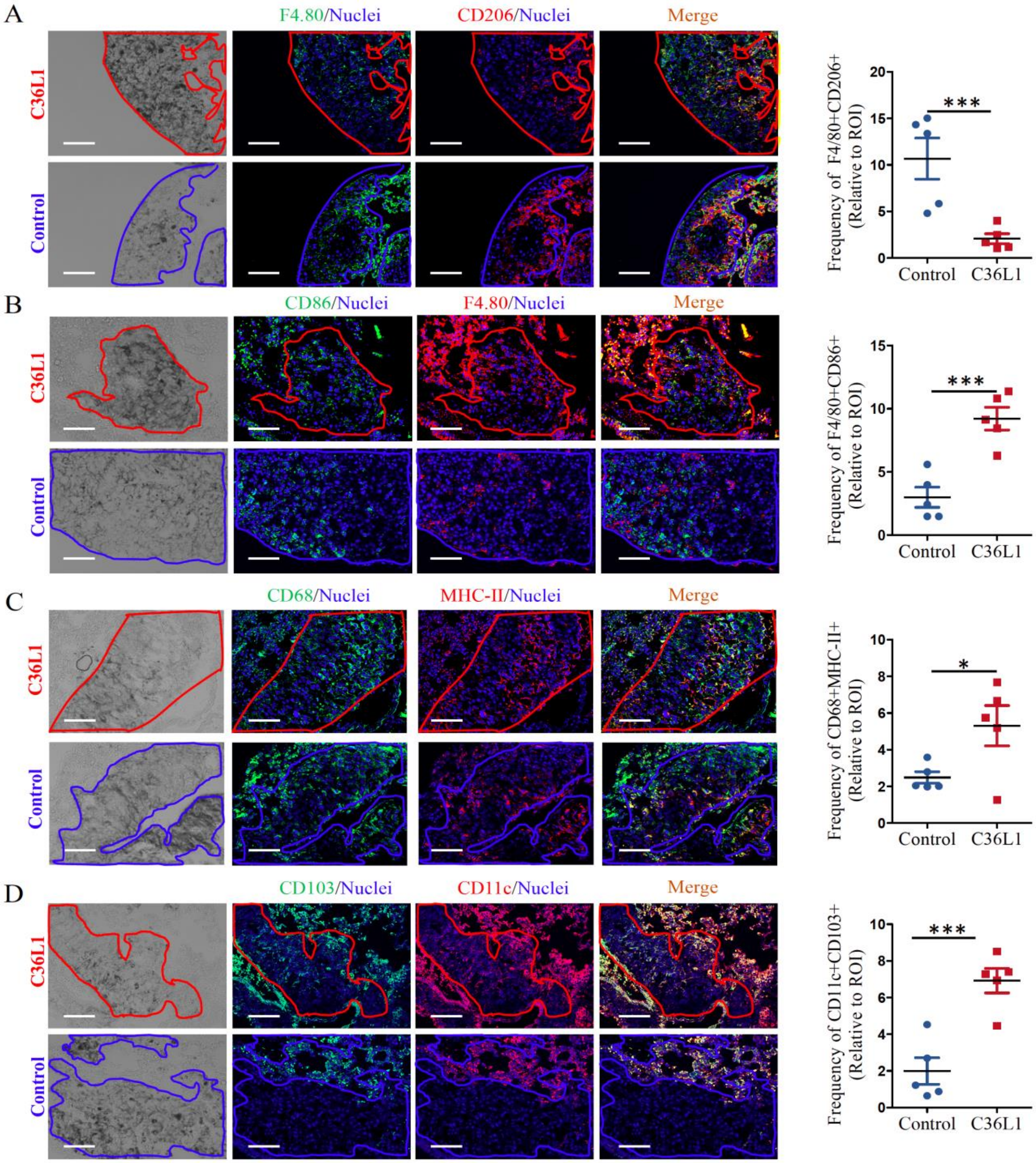

Supplementary Figure 1. Left, representative immunofluorescent images of different infiltrated immune cells in lung metastasis from C36L1 and control vehicle treated mice. Melanoma lung metastatic area appears in dark/brown colour in brightfield images. Right, Graphs show quantification of **(A)** F4/80^{+}CD206^{+} M2-like (***p=0.0027), **(B)** F4/80^{+}CD86^{+} (***p<0.001) macrophages, (C) MHCII/CD68 M1-like macrophages, **(D)** CD11c^{+}CD103^{+} activated DCs (***p<0.001).

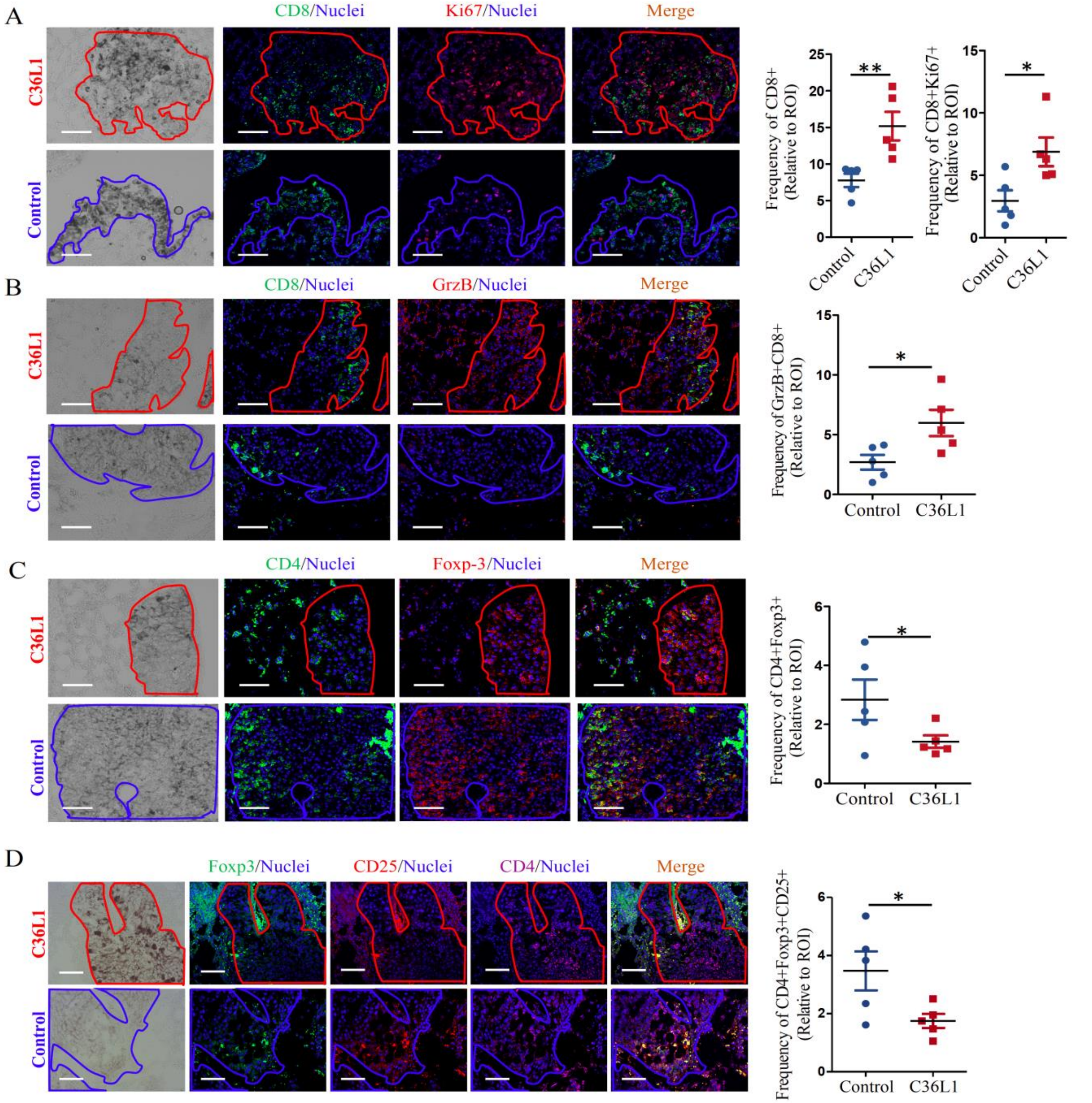

Supplementary Figure 2. Left, representative immunofluorescent images of different infiltrated immune cells in lung metastasis from C36L1 and control vehicle treated mice. Melanoma lung metastatic area appears in dark/brown colour in brightfield images. Right, Graphs show quantification of (A) $CD8^{+}granzymeB^{+}$ activated cytotoxic T cells, **(B)** $CD8^{+}Ki67^{+}$ proliferating cytotoxic T cells (*p=0.041), **(C)** $CD4^{+}Foxp3^{+}$ T regs (*p=0.015), (D) $CD4^{+}CD25^{+}FoxP3^{+}$ Tregs (*p=0.041). Nuclei were counterstained with Hoechst 33342 (Blue). Blue and red lines indicate the tumour area in C36L1 and control vehicle treated mice, respectively. Scale bars: 50 µm. *n* = 5 mice per group; at least three fields assessed per sample. Values are expressed as means ± s.e.m., and were analysed using a two-tailed unpaired *t*-test.

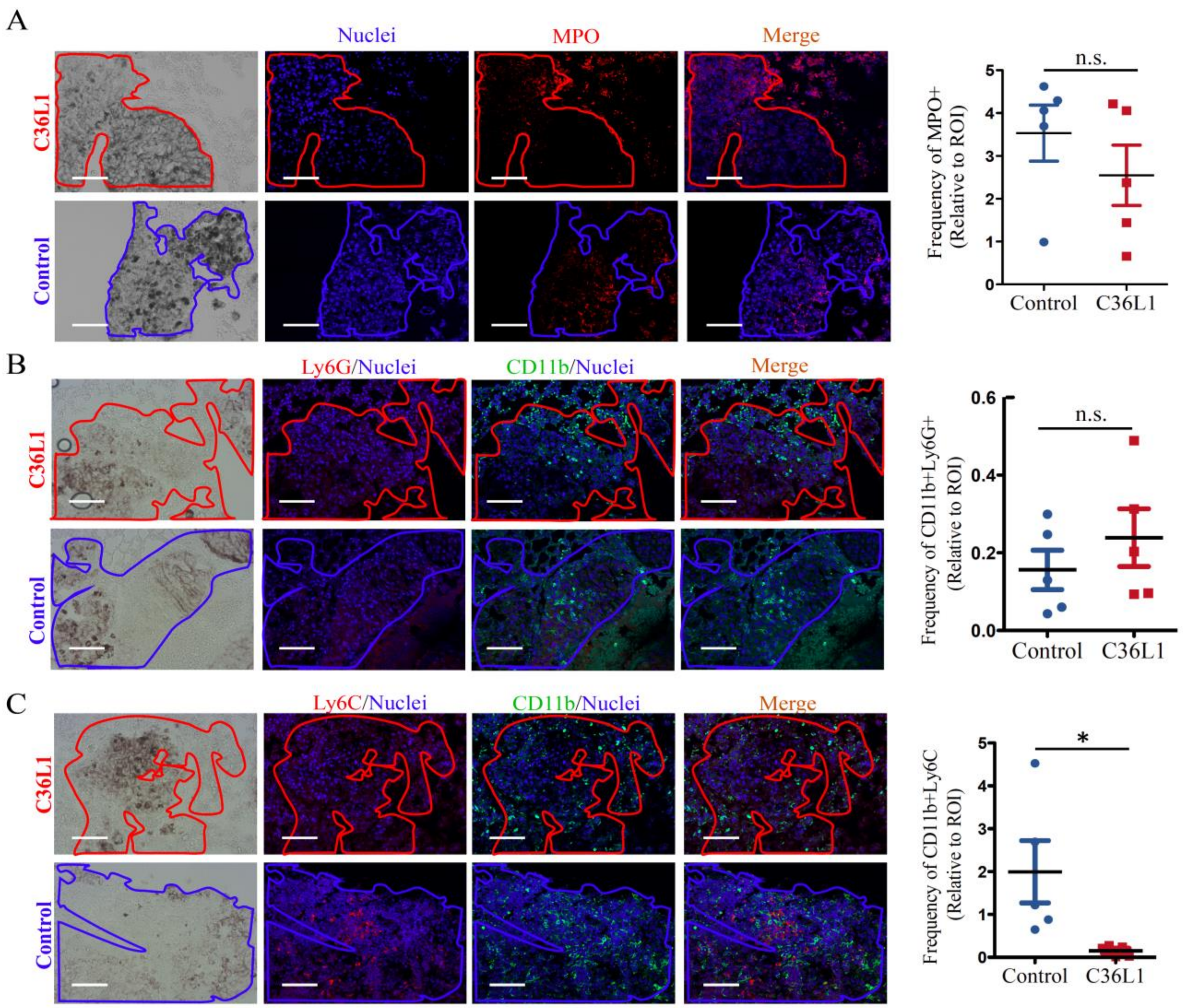

Supplementary Figure 3. (A) Immunofluorescent staining and quantification of myeloid-derived MPO^+ neutrophils in control and C36L1 treated metastatic tumours (n.s.; p=0.167). (B) Immunofluorescent staining and quantification of myeloid-derived polymorphonuclear suppressor cells $CD11b^+$ (Green) $Ly6G^+$ (red) in control and C36L1 treated metastatic tumours (n.s.; p=0.384). (C) Immunofluorescent staining and quantification of myeloid-derived monocytic suppressor cells $CD11b^+$ (green) $Ly6C^+$ (red) cells in control and C36L1 metastatic tumours (*p=0.035). n = 5 mice per group. Values are expressed as means ± s.e.m., and were analysed using a two-tailed unpaired t-test.

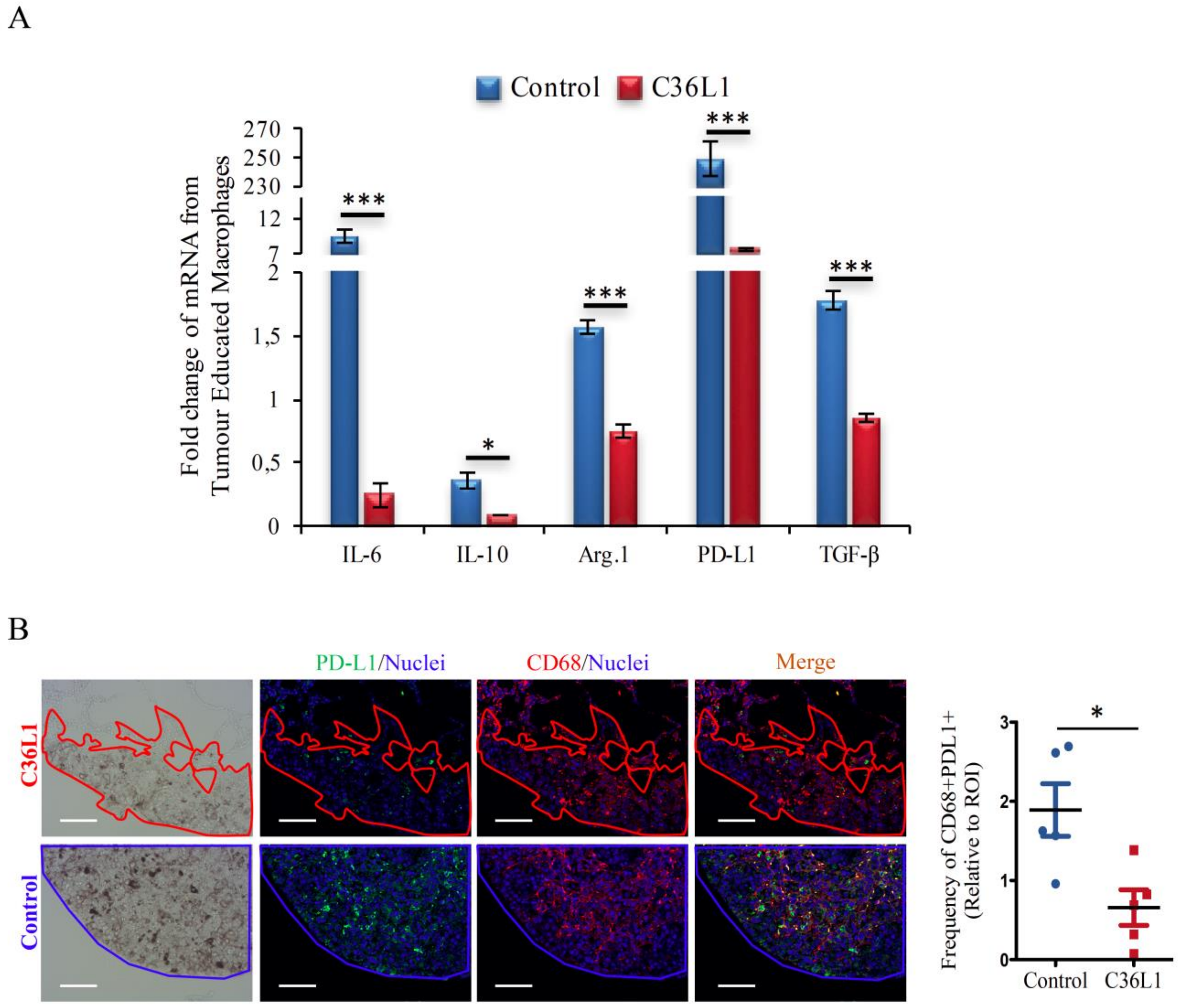

Supplementary Figure 4. (A) C36L1 treatment of tumour educated macrophages reduces the mRNA expression levels of the immunosuppressive factors TGF-β (***p < 0.001), IL-10 (*p=0.037), IL-6 (***p<0.001), Arg.1 (***p<0.001) and PD-L1 (**p=0.001). The experiment was performed with three biological replicates (n = 3). Values represent mean ± s.e.m and were analysed using a two-tailed unpaired *t*-test. (B) Quantification of $CD68^{+}PDL1^{+}$ metastasis associated macrophages in control and C36L1 treated tumours (*p=0.051). *n* = 5 mice per group; at least three fields assessed per sample. Values are expressed as means ± s.e.m., and were analysed using a two-tailed unpaired *t*-test.

A

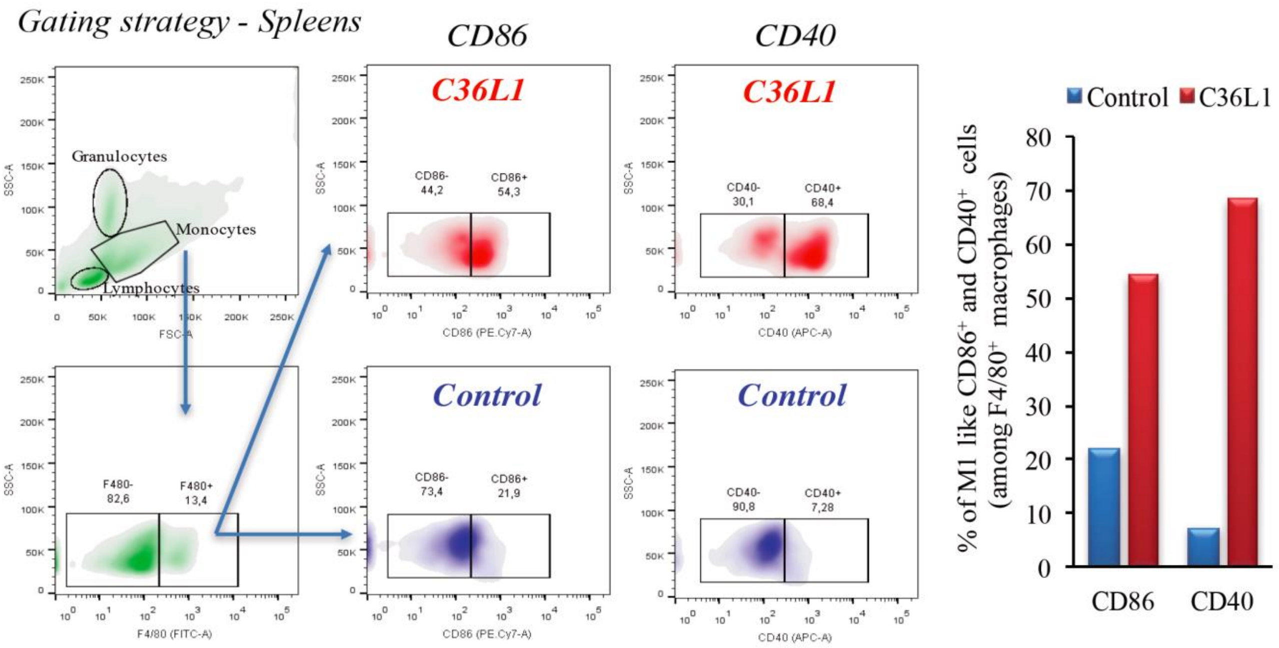
Gating strategy - Spleens
CD86
CD40
C36L1
Control
Granulocytes
Monocytes
Lymphocytes
SSC-A
FSC-A
F480-
82,6
F480+
13,4
F4/80 (FITC-A)
CD86-
44,2
CD86+
54,3
CD86-
73,4
CD86+
21,9
CD86 (PE.Cy7-A)
CD40-
30,1
CD40+
68,4
CD40-
90,8
CD40+
7,28
CD40 (APC-A)
Control
C36L1
% of M1 like CD86+ and CD40+ cells
(among F4/80+ macrophages)
CD86
CD40

B

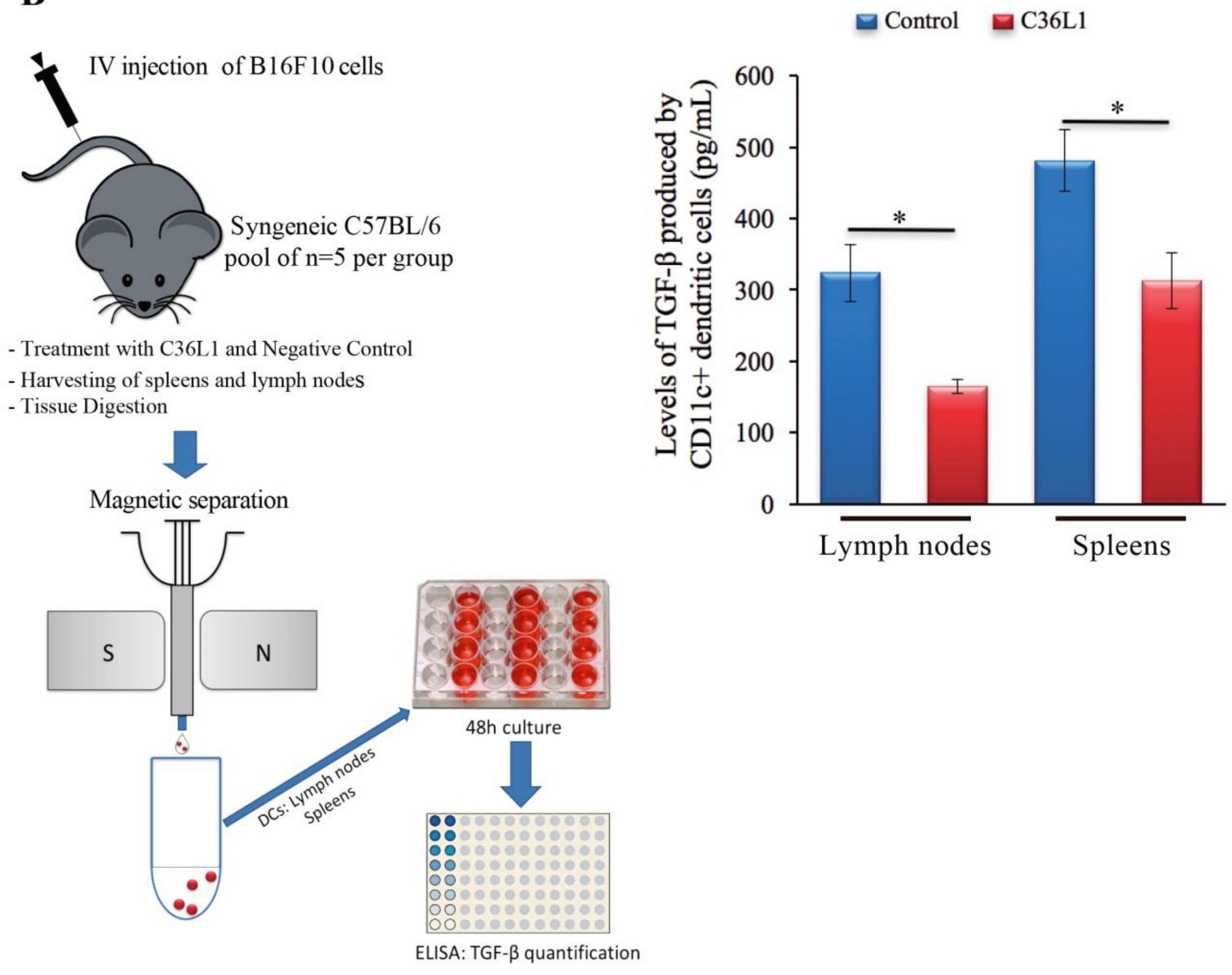
IV injection of B16F10 cells
Syngeneic C57BL/6
pool of n=5 per group
- Treatment with C36L1 and Negative Control
- Harvesting of spleens and lymph nodes
- Tissue Digestion
Magnetic separation
S
N
DCs: Lymph nodes
Spleens
48h culture
ELISA: TGF-β quantification
Control
C36L1
Levels of TGF-β produced by
CD11c+ dendritic cells (pg/mL)
*
Lymph nodes
Spleens

Supplementary Figure 5: (A) Left: FACS gating strategy and evaluation of $CD40^+$ and $CD86^+$ M1-Like markers among $F4/80^+$ macrophages from spleens of C36L1 and control vehicle mice. **Right:** Bar graphs represent absolute values from flow cytometry analysis of five pooled spleens per treatment group (control versus C36L1) from mice bearing metastatic melanoma tumours. Data represent one in vivo experiment with *n* = 5 mice per treatment group. **(B) Left:** Schematics describing the workflow and isolation of primary DCs harvested from lymph nodes and spleens of C36L1 and control treated mice. $CD11c^+$ DCs from each group (*n* = 5) were pooled together and incubated for 48h in triplicates. **Right:** Quantification of TGF-β levels secreted by DCs from spleens and cervical lymph nodes of C36L1 and control treated mice. Cytokine levels were determined by ELISA assay. Bar graphs represent mean ± s.e.m of [TGF-β] values from triplicates. Data were analysed using a two-tailed unpaired t-test. Lymph nodes *p=0.018; Spleens *p=0.045.

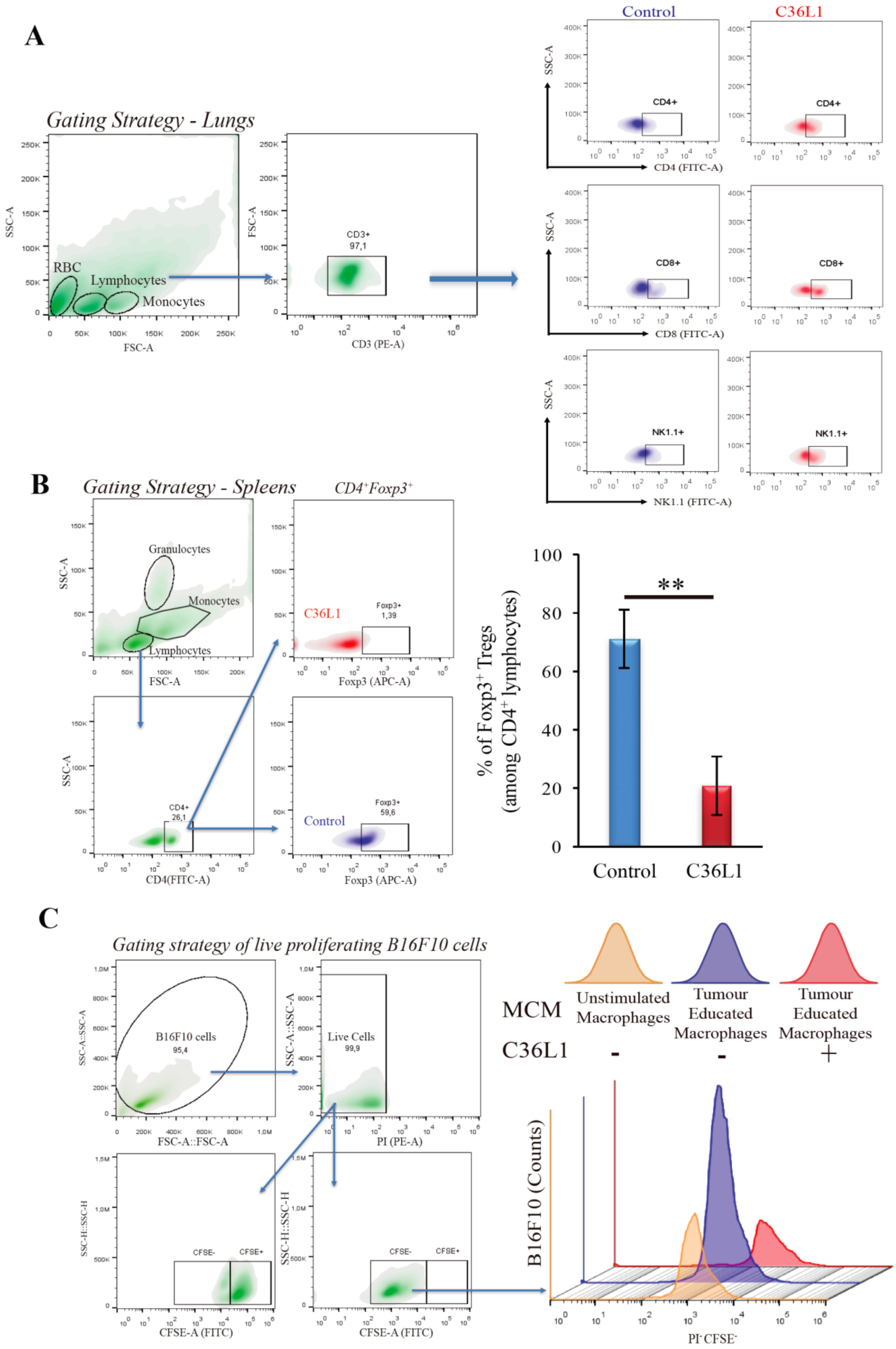
A
Gating Strategy - Lungs
RBC
Lymphocytes
Monocytes
SSC-A
FSC-A
CD3+
97,1
CD3 (PE-A)
Control
C36L1
CD4+
CD4 (FITC-A)
CD8+
CD8 (FITC-A)
NK1.1+
NK1.1 (FITC-A)
B
Gating Strategy - Spleens
CD4+Foxp3+
Granulocytes
Monocytes
Lymphocytes
CD4+
26,1
CD4(FITC-A)
Foxp3+
1,39
Foxp3+
59,6
Foxp3 (APC-A)
% of Foxp3+ Tregs (among CD4+ lymphocytes)
**
Control
C36L1
C
Gating strategy of live proliferating B16F10 cells
B16F10 cells
95,4
FSC-A::FSC-A
SSC-A::SSC-A
Live Cells
99,9
PI (PE-A)
SSC-H::SSC-H
CFSE-
CFSE+
CFSE-A (FITC)
MCM
Unstimulated Macrophages
Tumour Educated Macrophages
Tumour Educated Macrophages
C36L1
-
-
+
B16F10 (Counts)
PI- CFSE-

Supplementary Figure 6: (A) Representative flow cytometry density plots showing gating strategy used to quantify lung infiltrated T cells ($CD3^{+}CD8^{+}$, $CD3^{+}CD4^{+}$ and $CD3^{+}NK1.1^{+}$). **(B) Left**, representative flow cytometry density plots showing the levels of $CD4^{+}Foxp3^{+}$ Tregs in spleens of C36L1 and control vehicle treated mice. **Right**, bar graph represents mean ± s.e.m of flow cytometry data combined from 3 independent in vivo experiments. *n* = 5 pooled mice per group. Data were analysed using a two-tailed unpaired t-test **p = 0.0089. **(C) Left**, gating strategy used to quantify B16F10 cells proliferation by flow cytometry. The number of proliferating ($CFSE^{-}$) B16F10 cells is quantified among live cells (PI^{-}). **Right**, histograms represent counts of proliferating cells.

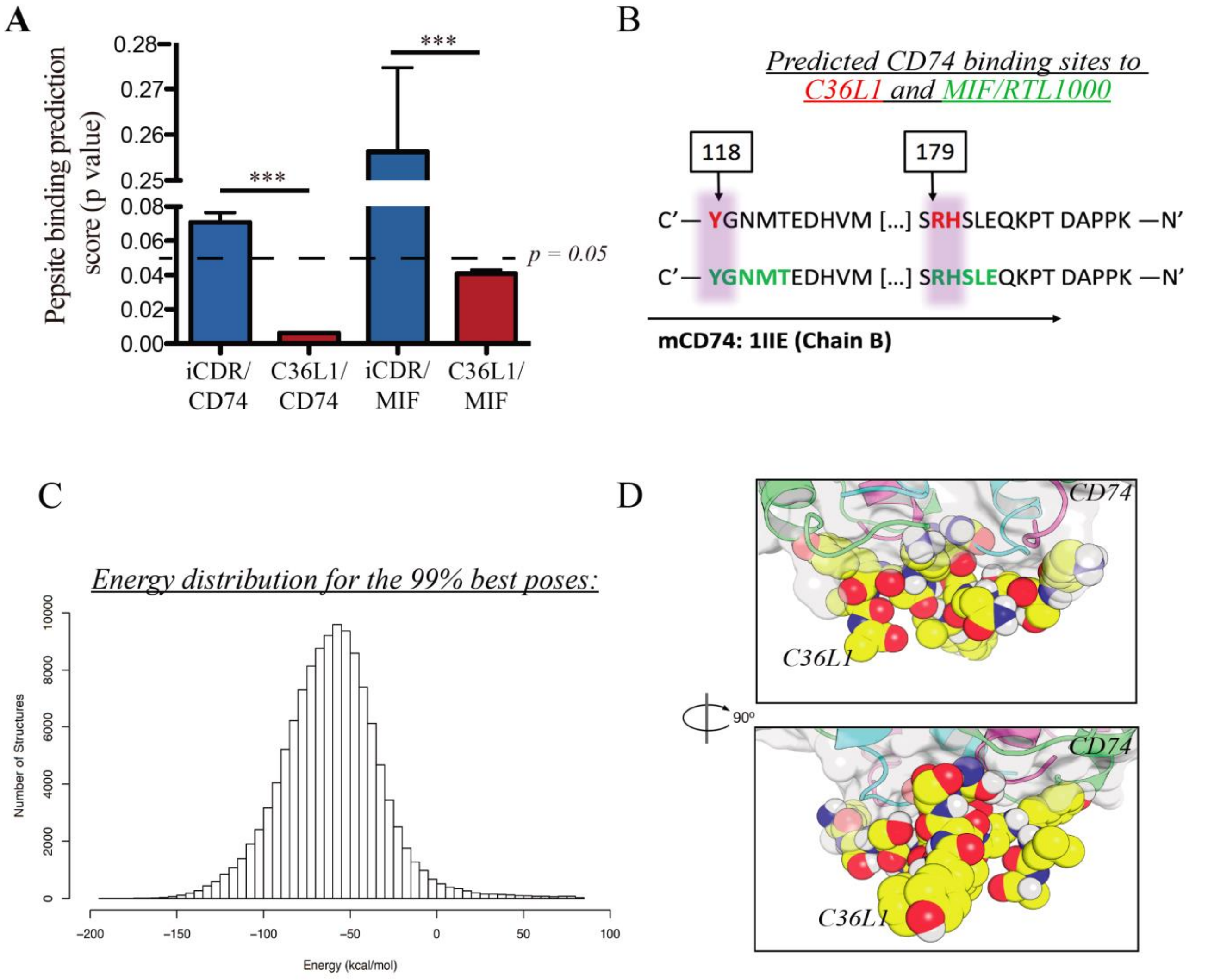

Supplementary Figure 7: (A) Binding prediction of C36L1 and iCDR peptides to CD74 receptor using Pepsite 2.0 algorithm. Bar graph represents mean ± s.e.m of p values. (n = 5, top best rank predictions). Data were analysed using a two-tailed unpaired t-test (***p<0.001). **(B)** Prediction of specific aminoacid residues in C36L1 interacting with CD74. Murine CD74 chain B (PDB: 1IIE) residues that interact with C36L1 (in red) and MIF/RLT1000 (in green) are shown. **(C)** Energy histogram distribution regarding the 99% best poses of CD74 complexed with C36L1. Normal mode 10 are found in both histogram ends. Energy poses at the negative end correspond to a larger displacement along the mode while energy poses at the positive end are more closely related to the minimized conformation. **(D)** Steric complementarity between partners. CD74 surface is shown in gray and C36L1 atoms are represented by their van der Waals radius.

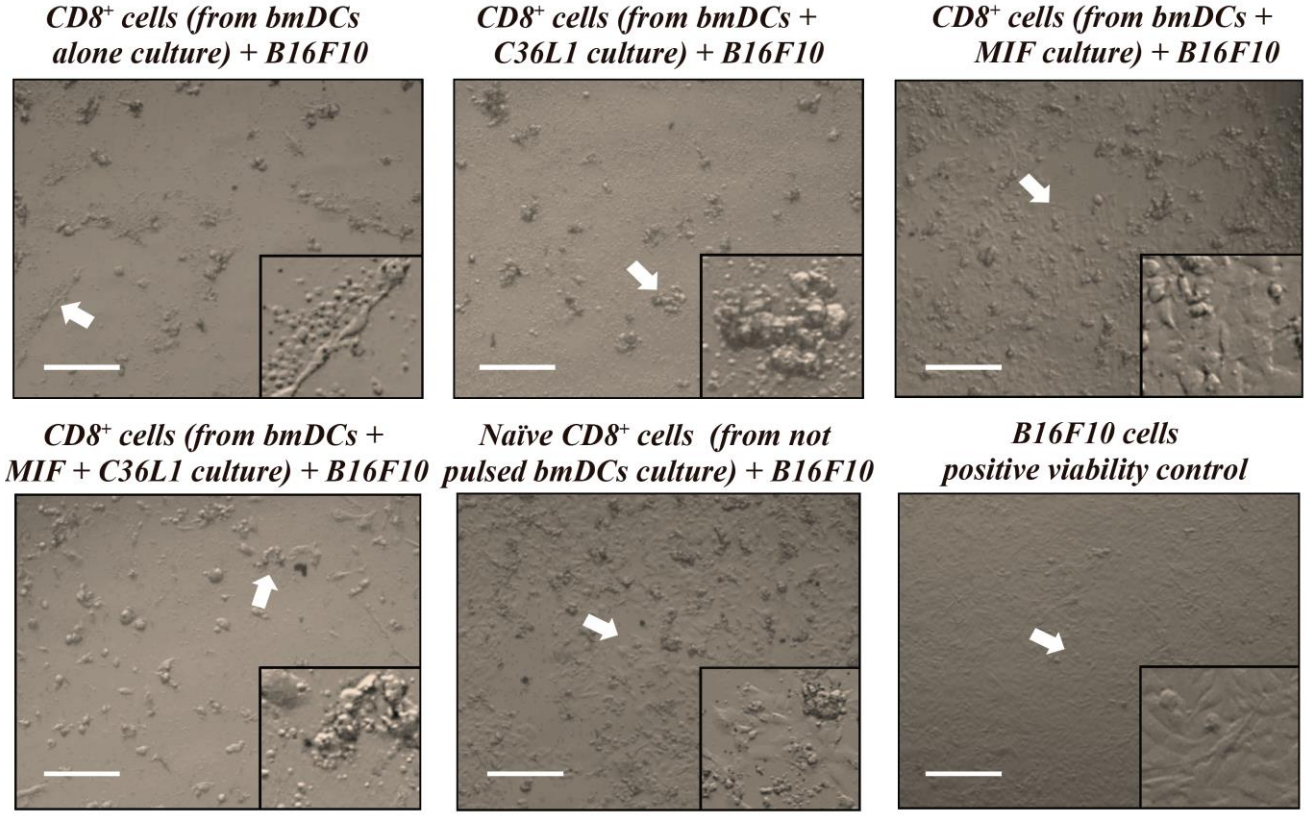

Supplementary Figure 8: Bright field microscopy representative images from co-cultured B16F10 cells and $CD8^+$ T cells. White arrows indicate inserts highlighting $CD8^+$ T cells killing B16F10 cells. Scale bars 50 μm.
